# B′-protein phosphatase 2A is a functional binding partner of delta-retroviral integrase

**DOI:** 10.1093/nar/gkv1347

**Published:** 2015-12-10

**Authors:** Goedele N. Maertens

**Affiliations:** Division of Infectious Diseases, St. Mary's campus, Imperial College London, Norfolk Place, London, W2 1PG, UK

## Abstract

To establish infection, a retrovirus must insert a DNA copy of its RNA genome into host chromatin. This reaction is catalysed by the virally encoded enzyme integrase (IN) and is facilitated by viral genus-specific host factors. Herein, cellular serine/threonine protein phosphatase 2A (PP2A) is identified as a functional IN binding partner exclusive to δ-retroviruses, including human T cell lymphotropic virus type 1 and 2 (HTLV-1 and HTLV-2) and bovine leukaemia virus (BLV). PP2A is a heterotrimer composed of a scaffold, catalytic and one of any of four families of regulatory subunits, and the interaction is specific to the B′ family of the regulatory subunits. B′-PP2A and HTLV-1 IN display nuclear co-localization, and the B′ subunit stimulates concerted strand transfer activity of δ-retroviral INs *in vitro*. The protein–protein interaction interface maps to a patch of highly conserved residues on B′, which when mutated render B′ incapable of binding to and stimulating HTLV-1 and -2 IN strand transfer activity.

## INTRODUCTION

Of the seven retroviral genera (lenti-, α-, β-, γ−, δ-, ϵ- and spumavirinae), two are known to cause severe and fatal conditions in humans. The lentiviruses human immunodeficiency virus type 1 (HIV-1) and HIV-2 are the aetiological agents of acquired immunodeficiency syndrome. The δ-retrovirus human T-cell lymphotropic virus type 1 (HTLV-1) causes adult T cell leukaemia-lymphoma (ATL) and the neurological disorder HTLV-1 associated myelopathy/tropic spastic paraparesis (HAM/TSP). A range of inflammatory diseases, such as uveitis, infective dermatitis, arthritis and others is also caused by HTLV-1 infection. Approximately 5% of HTLV-1 infected people eventually develop ATL, of whom most die within two years of symptom presentation (1). The treatment of both the inflammatory and malignant conditions remains unsatisfactory.

To establish infection, a retrovirus must insert a DNA copy of its RNA genome into host cell DNA. The key steps of this process are catalysed by the retroviral enzyme integrase (IN). Following reverse transcription, IN recognizes and binds to the viral long terminal repeat (LTR) ends of the viral DNA in a sequence specific manner to form the pre-integration complex (PIC) (2). Within the PIC, IN cleaves the 3′ ends of the viral DNA adjacent to invariant CA dinucleotides. This step, known as the 3′ processing reaction, produces 3′-OH groups that are used as nucleophiles during strand transfer. Following engagement with host DNA into a target capture complex, the 3′-OH groups attack scissile phosphodiester bonds on opposing strands within the target DNA, splicing the viral and cellular genomes together. The resulting single strand gaps in the integration intermediate are repaired by cellular machinery to yield the integrated provirus flanked by a short duplication of host DNA, which is 6 bp for the δ-retroviruses (3,4). Whilst the interaction between IN and viral LTR ends is DNA sequence-specific (5), the engagement of host DNA is much less so (6). Alignments of experimentally determined integration sites uncovered weakly conserved palindromic DNA consensus sequences (3,6–10). On the genomic level, integration site selection is determined by the interaction between IN and genus-specific host factors. Indeed, INs from HIV-1 and other lentiviruses bind LEDGF/p75, a transcriptional co-activator that targets PICs to actively transcribed genes (11–13), whilst γ-retroviral INs usurp a family of bromodomain containing proteins to home in on transcription start sites (14–16).

Recent high-throughput genome-wide sequencing of HTLV-1 integration sites from HAM/TSP patients combined with *in vitro* generated integration sites revealed a strong bias for integration into active regions of the genome (3). In a subset of the analysed integration sites, there appeared to be a strong preference for integration within close proximity of certain transcription factor binding sites (TFBS) (17), a trait that is shared by HTLV-2 (4). The strongest integration site preference was observed near p53, STAT1 and HDAC6 TFBS (17). Based on these observations, it seems unlikely that δ-retroviral PICs associate with a single transcription factor/chromatin binding protein, but rather might be targeted to the site of integration by a protein that associates with different transcription factors/chromatin binding proteins.

Herein, proteomic analyses identified a heterotrimeric serine/threonine protein phosphatase PP2A as a binding partner of δ-retroviral IN proteins. PP2A is a ubiquitously expressed protein involved in a myriad of cellular processes (18). PP2A is active as a heterotrimer; the core dimer composed of the catalytic C subunit and the scaffold subunit A is joined by one of four different regulatory subunit families: B, B′, B″ or B′″ to form the heterotrimer. Two highly conserved isoforms (α and β) exist for both the catalytic and the scaffold subunit. The B′ family is the largest, counting at least five members (α, β, γ, δ and ϵ) of which γ and δ express at least three isoforms, and ϵ has an additional alternative translation isoform. Formation of the holoenzyme regulates subcellular localization of PP2A, substrate specificity but also stability of the PP2A components (19).

Here, I show that the PP2A comprising the B′ regulatory subunits (hereafter referred to as B′-PP2A) associate specifically and exclusively with INs from the δ-retroviral genus, and the B′ subunits stimulate biologically-relevant concerted integration activity of HTLV-1 and -2 INs. Mapping of the interaction binding site on B′ illustrates that residues critical for binding to and stimulating HTLV-1 and -2 IN are highly conserved.

## MATERIALS AND METHODS

A detailed description of the generation of the DNA constructs and the Methods describing the purification of all recombinant proteins used in this manuscript, the stable HEK293T-derived cell lines generated and conditions for immunoprecipitation and nickel-nitrilotriacetic acid (Ni-NTA) pull-downs can be found in the Supplementary Data.

### Strand transfer assays

Short double stranded donor DNAs that mimic the U5 end of the HTLV-1 or HTLV-2 LTR were made by annealing oligonucleotides shown in Supplementary Table S1. For HTLV-1, blunt DNA substrate was made by annealing S20B with S20UN, whilst donor DNA mimicking 3′ processed LTR ends was made by annealing S20B with S20UP (20). For HTLV-2 IN substrate DNA of different lengths were used; donor DNA of 24 nucleotides mimicking pre-processed DNA was made by annealing S24UP with S24B whilst blunt DNA substrate was produced by annealing S24B with S24UN. Similarly, donor DNA counting 19 or 30 base pairs were made by annealing S19B with S19UP (3′ processed mimic) or S19UN (blunt) and S30B with S30UP (3′ processed mimic) or S30UN (blunt). Strand transfer reactions contained 0–4 μM B′γ(11–380), 2.8 μM donor DNA, 50 mM NaCl, 10 mM MgCl_2_, 13 mM DTT, 5.8 μM ZnCl_2_, 0.132 M HEPES pH 7.1 and 300 ng pGEM-9Zf(-) in a total volume of 30 μl and were started by addition of IN to a final concentration of 1 μM to 6 μM. B′γ(11–380) was used at different concentrations as indicated in the figures. Reactions, which spanned 60 or 90 min as indicated in figure legends, were stopped by addition of 0.5% SDS/25 mM EDTA, pH 8.0. Proteins were degraded by incubation with 30 μg proteinase K at 37°C for 1 h. DNA products, concentrated by ethanol precipitation, were separated by electrophoresis through 1.5% agarose and detected by staining with GelRed (VWR). To quantify strand transfer by PCR, donor DNA S20PQ (made by annealing S20UPQ with S20BQ) for HTLV-1 and S24PQ (by annealing S24UPQ with S24BQ) for HTLV-2 IN were used. Quantification was done as described previously (21). Standard curves were made by serial dilution of a reaction supplemented with wild type B′γ(11–380). Concerted integration products were isolated and cloned for sequencing as described previously (6,22,23).

## RESULTS

### Identification of δ-retroviral IN specific binding partners

**Figure 1.**
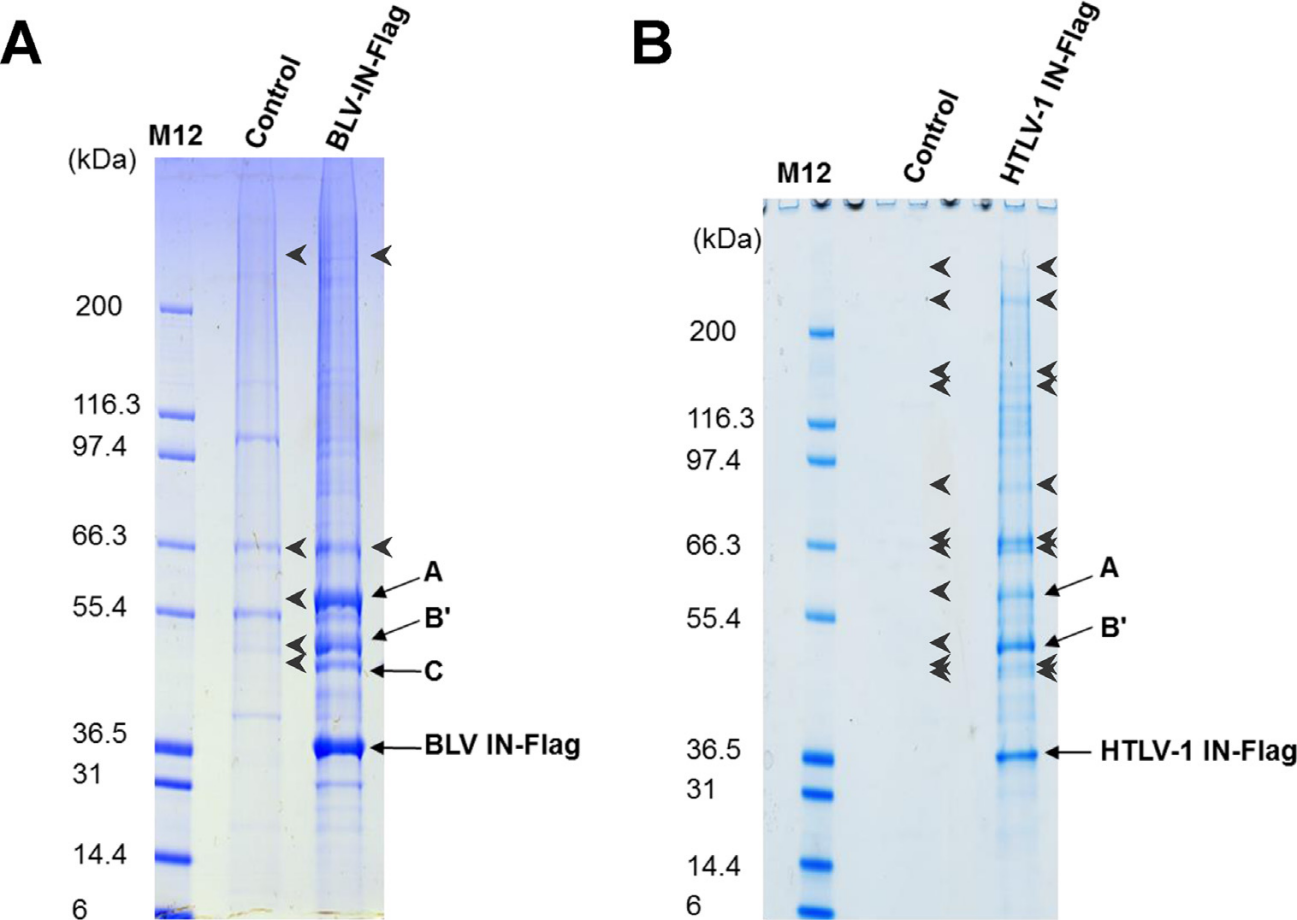
PP2A co-precipitates with BLV and HTLV-1 IN. Colloidal coomassie stained gels of the large scale Flag-IPs done with BLV IN (**A**) and HTLV-1 IN (**B**). Bands from the IN and the negative control lanes (indicated with arrows and arrow heads) were excised and analysed in parallel. Migration of the PP2A components is indicated to the right of the gel. Migration of the protein standard (Mark 12, Life Technologies) in kDa is indicated to the left of the gels.

**Figure 2.**
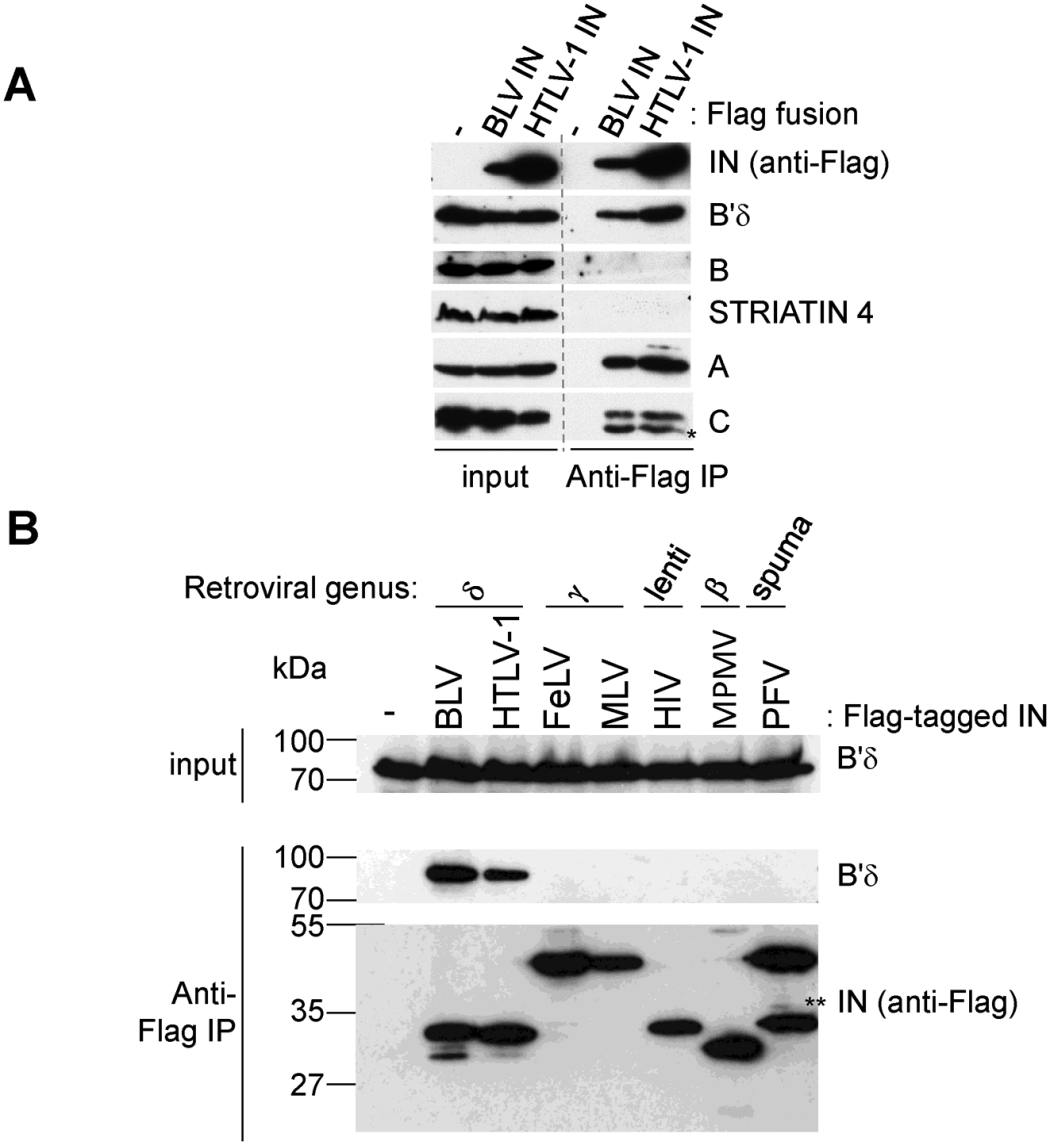
The interaction between PP2A and IN is genus-specific. (**A**) Flag-IP of transiently expressed Flag-tagged BLV and HTLV-1 IN in HEK293T cells. This is not a composite image; the vertical line was added to delineate the border between input and IP samples. *Degradation product of Cα. (**B**) Flag-IP using INs from different retroviral genera stably expressed in HEK293T cells. As a negative control, the parental cell line was used. Retroviral genus is indicated above the blot. Antibodies used are indicated to the right of the blot. **Degradation product of PFV IN.

To identify specific binding partners of δ-retroviral INs, proteins co-purifying with HTLV-1 or BLV IN (bovine leukaemia virus integrase) from extracts of HEK293T cells stably expressing C-terminally Flag-tagged versions of the viral proteins were analysed by tandem mass spectrometry. Several transcription factors, chromatin remodellers, enzymes involved in DNA damage repair as well as subunits of the cohesin and condensin complexes were isolated in these experiments (Supplementary Tables S2 and S3). Strikingly, subunits of the heterotrimeric phosphatase PP2A co-purified with BLV IN in close to stoichiometric quantities (Figure 1) including α, γ, δ and ϵ isoforms of its B′ regulatory subunit (Supplementary Table S2). Peptides corresponding to the scaffold and B′ regulatory subunits were also highly enriched with HTLV-1 IN (Figure 1, Supplementary Table S3). Absence of the brain-specific β isoform of B′ was not unexpected (24,25). In contrast to B′ isoforms, no peptides derived from the B and B″ regulatory PP2A subunits were found in the immunoprecipitates. Western blotting confirmed enrichment of the catalytic, scaffold and B′ subunits, and the absence of a B-type regulatory subunit in HTLV-1 IN-Flag immunoprecipitations (IPs) (Figure 2A). Although 4 peptides were identified from STRIATIN 4 (a B′″ type of regulatory subunit, Supplementary Table S3), interaction between BLV or HTLV-1 IN and STRIATIN 4 could not be confirmed by independent Flag-IPs (Figure 2A). The HTLV-1 IN-B′ interaction resisted challenge with NaCl concentrations up to at least 450 mM (Supplementary Figure S1).

To investigate whether PP2A is a binding partner of retroviral INs in general, or specifically associates with δ-retroviral INs, IPs were done with extracts from cells that stably express C-terminally Flag-tagged versions of HIV-1 (lentivirus), feline leukaemia virus (FeLV, γ-retrovirus), murine leukaemia virus (MLV, γ-retrovirus), prototype foamy virus (PFV, spumaretrovirus), Mason Pfizer monkey virus (MPMV, β-retrovirus), BLV and HTLV-1 IN (δ-retrovirus) versus the parental HEK293T cell line. B′-PP2A co-purified specifically and exclusively with IN proteins from the δ-retroviral genus (Figure 2B). Immunostaining of transiently expressed B′γ(11–380) fused to enhanced green fluorescent protein (EGFP-B′γ(11–380)) and Flag-tagged IN in HeLa cells showed that B′γ(11–380) co-localizes with HTLV-1 IN in the nucleus (Supplementary Figure S2). Although PFV IN was also nuclear, it displayed a more punctate distribution pattern clearly distinct from that of B′γ(11–380) (Supplementary Figure S2). Since monomeric B′ subunits are degraded rapidly in cells, localization of B′ subunits reflects the intracellular distribution of B′ containing PP2A holoenzymes (26), suggesting that HTLV-1 IN co-localizes with B′-PP2A.

### PP2A associates with δ-retroviral IN directly through the B′ regulatory subunit

**Figure 3.**
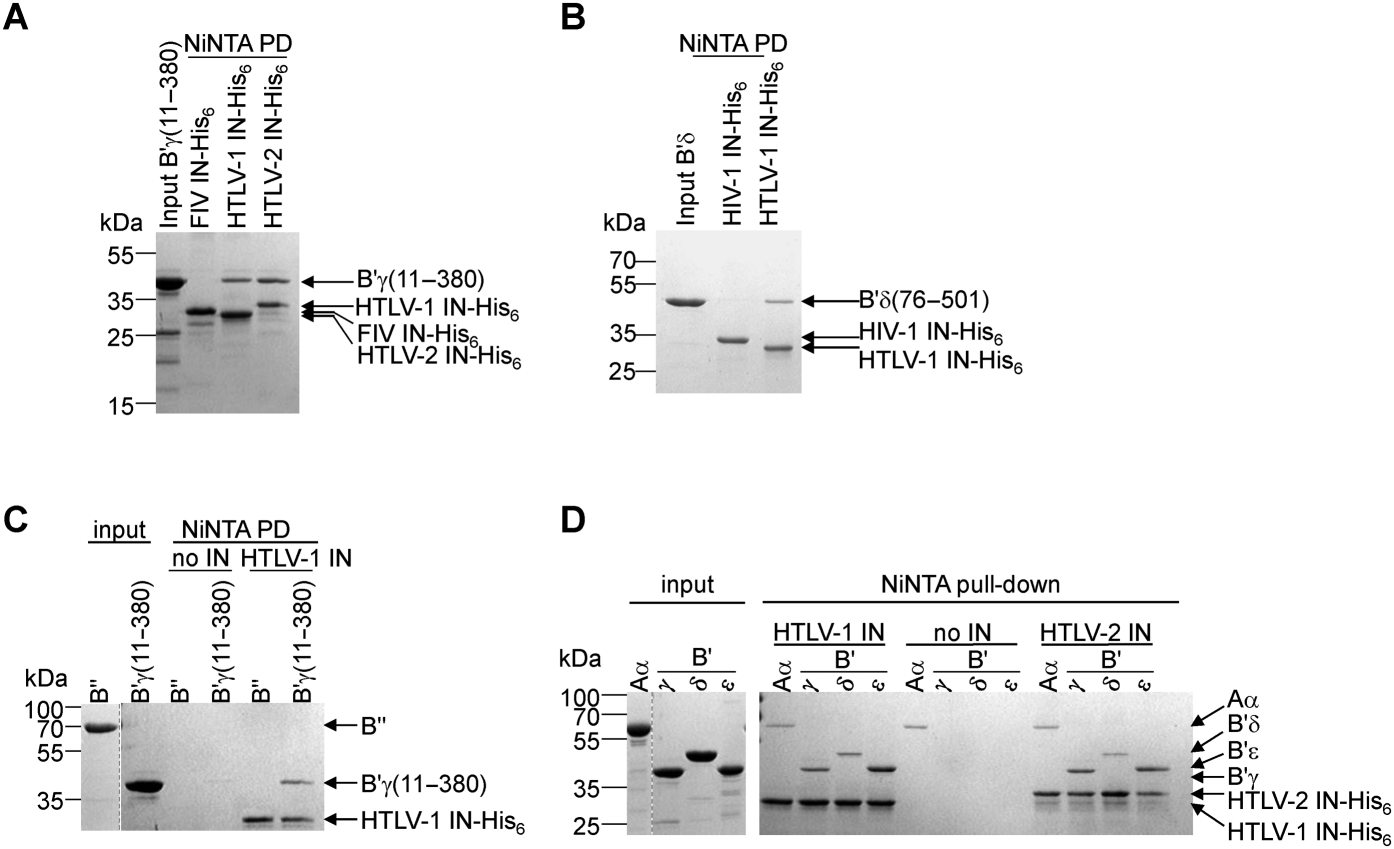
HTLV-1 IN binds directly to the B′ regulatory subunit. (**A**) NiNTA pull-downs using hexahistidine tagged HTLV-1 and -2 or FIV IN as bait. Five micrograms of bait and B′γ(11–380) were used. Input and pull-down samples are indicated above the gel. (**B**) B′δ(76–501) binds to HTLV-1 IN but not to HIV-1 IN. NiNTA pull-downs as described in (A). Migration of B′δ(76–501) and the IN proteins is indicated to the right of the gel. Lane 1: input of B′δ(76–501), lane 2: pull-down using HIV-1 IN-His_6_ as bait, lane 3: pull-down using HTLV-1 IN-His_6_ as bait. (**C**) NiNTA pull-down with either no IN (negative control) or HTLV-1 IN-His_6_ as bait. Addition of B″, or B′γ(11–380) to the pull-downs is indicated above the gel. The input of B″ was originally loaded on a different gel; the vertical line illustrates the border between the two gels. (**D**) Three isoforms of B′: B′γ(11–380), B′δ(76–501) and B′ϵ(51–401), and the scaffold subunit Aα were tested for binding to HTLV-1 and HTLV-2 IN-His_6_ by NiNTA pull-down. No IN was added in the negative control samples. The input of Aα was originally loaded on a different gel; the vertical line illustrates the border between the two gels. Input and pull-down are indicated above the gel. Migration of the proteins is indicated to the right of the gels. Migration of the molecular weight marker (in kDa) is indicated to the left of the gel. All gels were stained with Coomassie.

Since only members of the B′ family and none from the other families of regulatory subunits specifically co-purified with both the HTLV-1 and BLV IN Flag-IPs, the regulatory subunit likely mediates the interaction. Given the lack of sequence homology with HEAT (*h*untingtin-*e*longation *A*-subunit-*T*OR) repeat proteins, the B′ proteins are referred to as pseudo-HEAT repeat proteins (27–29), in which pseudo-HEAT repeats (composed of two anti-parallel α-helices connected by intra-loops) are arranged in tandem and linked by short inter-unit loops. The B′ pseudo-HEAT repeats compose a highly conserved central region (with ∼80% amino acid sequence identity) (Supplementary Figure S3), whilst the distinct N-terminal and C-terminal extensions are thought to contribute to substrate specificity. The conserved central region of three B′ isoforms (γ, δ, ϵ), one full-length B″ regulatory subunit, and the α isoform of the scaffold subunit Aα were cloned, expressed and purified from bacteria. Ni-NTA pull down assays were performed using hexa-histidine-tagged IN proteins. Since only very low yields of soluble recombinant full-length BLV IN were recovered from bacteria, the δ-retroviral INs from HTLV-1 and HTLV-2 were used in these experiments; the lentiviral HIV-1 and feline immunodeficiency virus (FIV) INs served as negative controls. B′γ(11–380) (Figure 3A) and B′δ(76–501) (Figure 3B) interacted with HTLV-1 and HTLV-2 IN, but not with the lentiviral INs. Moreover, only the B′ isoforms directly associated with HTLV-1 and HTLV-2 IN, as direct association between HTLV-1 IN and B″ was not observed (Figure 3C and D). B′ϵ(51–401) interacted with HTLV-1 and -2 IN, whilst the structural subunit, Aα, appeared to non-specifically precipitate on the Ni-NTA beads (Figure 3D).

### B′ stimulates concerted strand transfer activity of δ-retroviral INs

**Figure 4.**
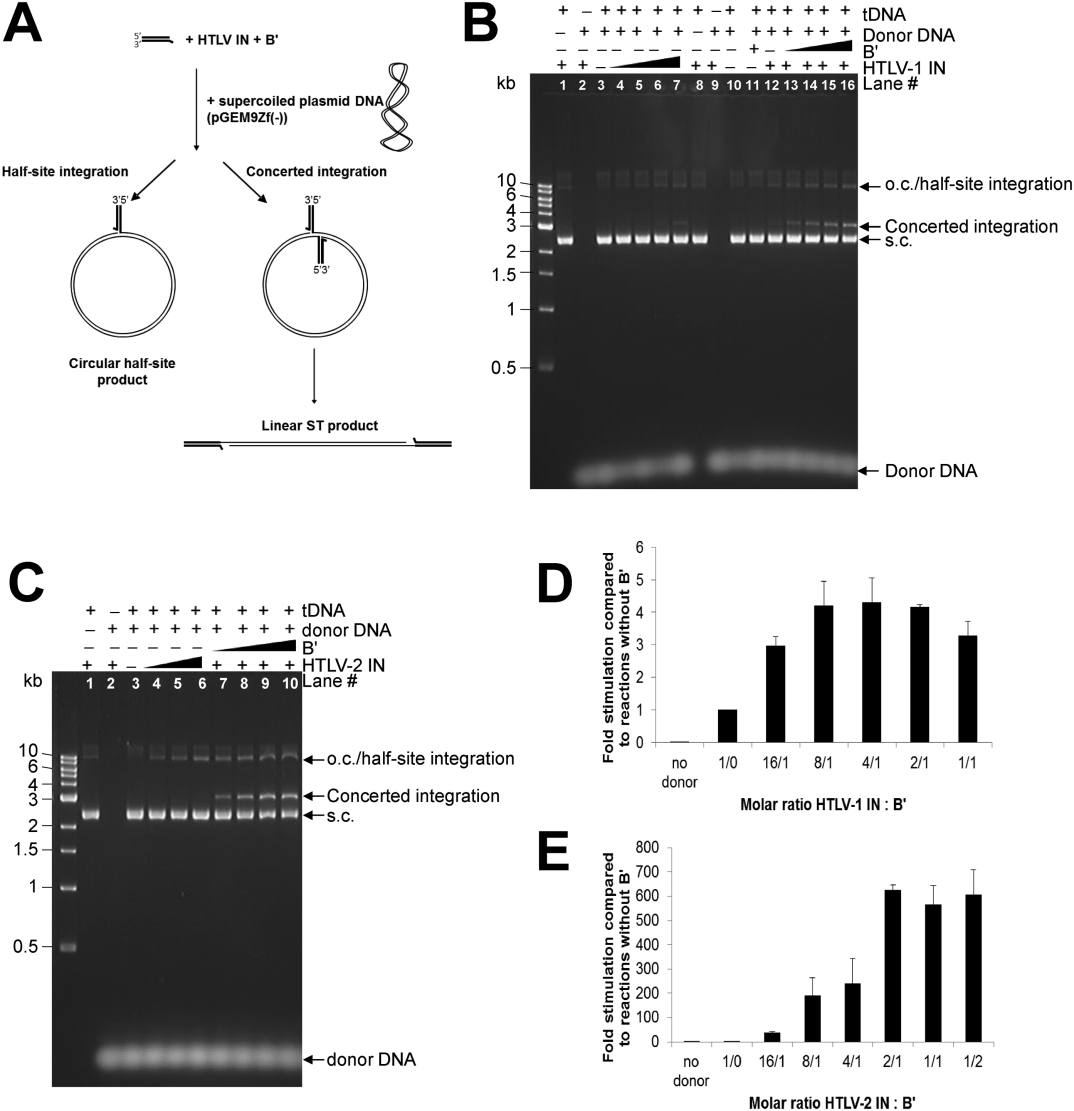
B′γ stimulates δ-retroviral strand transfer activity of HTLV-1 and -2 IN. (**A**) Scheme explaining concerted versus half-site integration (23). Whereas insertion of one donor DNA substrate into the supercoiled plasmid DNA results in an open circular form of DNA (half-site integration) (left), concerted integration of two donor DNA molecules results in a linear strand transfer product (right). (B) and (C) Donor DNA mimicking the 3′-processed ends was used (S20P for HTLV-1 IN and S30P for HTLV-2 IN). Presence of donor DNA, B′γ(11–380), HTLV-1 IN (B) or HTLV-2 IN (C) and tDNA (pGEM9zf(-)) are indicated above the gel. (**B**) Integration assays with HTLV-1 IN. Concentration of proteins used: in the absence of B′γ(11–380): lanes 1, 2, 8, 9 and 12: 4 μM IN; lanes 3 and 10: no IN; lane 4: 1 μM IN; lane 5: 2 μM IN; lane 6: 4 μM IN; lane 7: 6 μM IN. Lanes 12–16 were done in the presence of 4 μM IN with the following concentration of B′γ(11–380): lane 12: 0 μM B′; lane 13: 0.25 μM B′; lane 14: 0.5 μM; lane 15: 1 μM, lane 16: 2 μM. Lane 11 was done in the absence of IN and presence of 2 μM B′γ(11–380). (**C**) Integration assays with HTLV-2 IN. In the absence of B′γ(11–380): lanes 1 and 2: 4 μM IN; lane 3: no IN; lane 4: 1 μM IN; lane 5: 2 μM IN; lane 6: 4 μM IN. Lanes 7–10 were done using 1 μM HTLV-2 IN and the following concentrations of B′γ(11–380): lane 7: 0.25 μM; lane 8: 0.5 μM; lane 9: 1 μM and lane 10: 2 μM. DNA products were separated on a 1.5% agarose gel and stained with GelRed. Migration of DNA species in the gel is indicated on the right of the gel. S.c., supercoiled; o.c., open circular. The 1 kb DNA ladder (NEB, indicated on the left of the gel) was used as a reference. This is a representative of experiments repeated four times (HTLV-1 IN) and three times (HTLV-2 IN). For quantification, S20PQ (HTLV-1 IN) (**D**) and S24PQ (HTLV-2 IN) (**E**) were used as donor DNA. Strand transfer activity was quantified as published earlier (21). A standard curve was made using reactions done with HTLV IN: B′γ in a 2:1 molar ratio and strand transfer products were quantified in relation to this standard curve. Fold stimulation of integration activity was determined by calculating the increase in product compared to the reactions done in the absence of B′γ. Bar plots were made by calculating the averages and standard deviations of three independent experiments.

*In vitro* activities of HTLV-1 and HTLV-2 IN were previously characterised using radio-actively labelled oligonucleotides that mimicked the respective LTRs. Balakrishnan and Jonsson showed that the catalytic activity of HTLV-1 and -2 IN was highly sensitive to NaCl, and 3′-processing and strand transfer activities were inhibited by concentrations exceeding 100 mM (20,30). All our reactions were done in the presence of 50 mM NaCl. To distinguish half-site integration from the biologically relevant concerted integration activity, the previously reported assay was used in which short viral LTR mimics function as donor DNA and supercoiled plasmid (pGEM) as target DNA (6,22,23). Concerted integration hence results in linearisation of the plasmid, whilst half-site integration co-migrates with open circular plasmid DNA (Figure 4A). Some concerted strand transfer activity was observed in the presence of 6 μM HTLV-1 IN (Figure 4B lane 7), and when using 4 μM HTLV-1 IN the activity was considerably increased in the presence of as little as 0.25 μM B′γ(11–380) (Figure 4B compare lanes 6 and 13). Similarly, the biologically-relevant activity of HTLV-2 IN was significantly increased in the presence of B′. The effect of B′γ(11–380) addition to the reaction was dose-dependent (Figures 4B and C) and concerted integration or nicking activity was not observed when B′γ(11–380) was added alone (Figure 4B lane 11). Whilst maximum stimulation of concerted integration activity was observed using a ∼4:1 molar ratio of HTLV-1 IN : B′γ, and 2:1 molar ratio for HTLV-2 IN : B′γ, a significant stimulation could be observed at ratios of HTLV IN : B′γ of 16:1 (Figure 4B lane 13 and Figures 4D and 4E). The ∼150-fold greater stimulation of concerted integration activity of HTLV-2 IN by B′γ compared to HTLV-1 IN is due to the fact that ∼150-fold less products of integration are detected when HTLV-2 IN is used on its own. Of importance, in these reactions 1 μM HTLV-2 IN is used compared to 4 μM HTLV-1 IN. However, repeating the experiments with 1 μM HTLV-1 IN still produces ∼78 times more integration product than HTLV-2 IN (data not shown). It thus appears that HTLV-1 IN is more active than HTLV-2 IN and HTLV-2 IN is more dependent on B′γ than HTLV-1 IN under the conditions described here.

**Figure 5.**
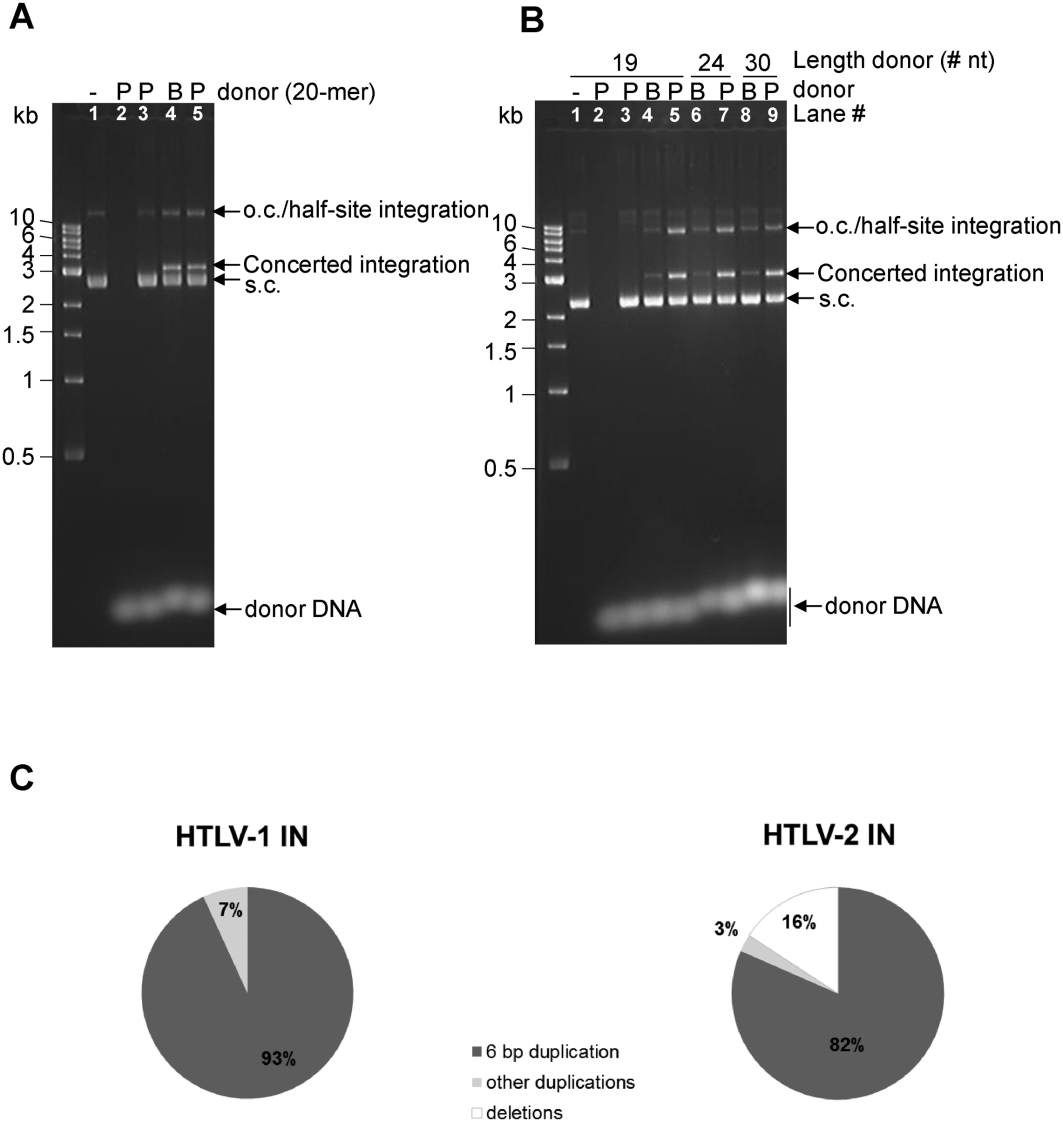
HTLV-1 and -2 IN are active on blunt substrates. Integration reactions done with HTLV-1 or HTLV-2 IN using either blunt (B) or pre-processed (P) donor DNA as substrate. Reactions were allowed to take place at 37°C for 90 min before the reactions were stopped. (**A**) Reactions done with HTLV-1 IN. All reactions (except for lane 3 where IN was omitted) contained 4 μM HTLV-1 IN and 2 μM B′γ(11–380). Lane 1: no donor, lane 2: no tDNA; lane 3: no IN; lane 4: blunt donor; lane 5: pre-processed donor. (**B**) Reactions with HTLV-2 IN. Donor DNA of different lengths were used as indicated above the gel. All reactions (except for lane 3 where IN was omitted) contained 1 μM HTLV-2 IN and 0.5 μM B′γ(11–380). Lane 1: no donor, lane 2: no tDNA; lane 3: no IN; lane 4: blunt donor DNA 19-mer; lane 5: pre-processed 19-mer; lane 6: blunt 24-mer; lane 7: pre-processed 24-mer; lane 8: blunt 30-mer and lane 9: pre-processed 30-mer. DNA products were separated on a 1.5% agarose gel and stained with GelRed. Migration of DNA species in the gel is indicated on the right of the gel. S.c. supercoiled; o.c., open circular. The 1 kb DNA ladder (NEB, indicated on the left of the gel) was used as a reference. Gels representative of three independent experiments are shown. (**C**) Products of concerted integration catalysed by HTLV-1 or -2 IN in the presence of B′ (2:1 molar ratio) were cloned and sequenced. Percentage of 6 bp duplication, other duplications and deletions are shown in pie charts. Fourty four clones were sequenced for HTLV-1 IN and 38 for HTLV-2 IN.

In the reactions described above, the substrates mimicked 3′ processed LTRs. When using blunt DNA substrates that represent the unprocessed LTRs and using HTLV-1 or -2 IN in the presence of B′γ using a 2:1 molar ratio, HTLV-1 IN appeared equally active for supporting concerted viral DNA integration (Figure 5A, compare lanes 4 and 5), whilst HTLV-2 IN was significantly more active on processed substrates (Figure 5B, compare lanes 4 and 5, 6 and 7, 8 and 9). Although four times as much HTLV-1 IN as HTLV-2 IN was used in these assays, the same was observed when using equivalent concentrations of HTLV-2 IN (Supplementary Figure S4, compare lanes 4 and 5). This suggests that formation of the active presynaptic complex is more efficient with HTLV-1 IN than with HTLV-2 IN.

Retroviral INs integrate the viral cDNA across the major groove of tDNA, attacking scissile phosphodiester bonds of opposing strands in a staggered fashion leading to a short duplication of the integration site sequence. Since no detectable (HTLV-2 IN) or very low levels (HTLV-1 IN) of concerted integration products are produced in the absence of the PP2A regulatory subunit, strand transfer products made in the presence of B′γ were cloned and sequenced as described previously (6,22,23). For HTLV-1, 41 (93%) of the cloned integration products had the expected 6-bp duplication, 2 clones a 7-bp (5%) and 1 clone had a 4-bp duplication (2%). Thirty one (82%) of the HTLV-2 IN concerted integration products had a 6-bp duplication, 1 clone (3%), a 5-bp duplication and 6 clones (16%), a deletion or rearrangement (Figure 5C). The small percentage of clones with deletions or rearrangements could possibly have arisen from the cloning or selection procedures used. Overall, the large majority of the HTLV-1 and -2 concerted integration products are legitimate. To investigate whether HTLV-1 or -2 IN influence the enzymatic activity of PP2A, Flag-tagged B′γ-PP2A holoenzymes were isolated from HEK293T cells (Supplementary Figure S5A and B) and phosphatase activity was measured using the phospho-Thr PP2A specific peptide (R-K-pT-I-R-R) (31). Only when 5-fold molar excess of HTLV-1 or -2 IN was added to the reactions was the Flag-tagged B′γ-PP2A activity reduced with ∼25–30% (Supplementary Figure S5C).

### Mapping of the interaction on B′

**Figure 6.**
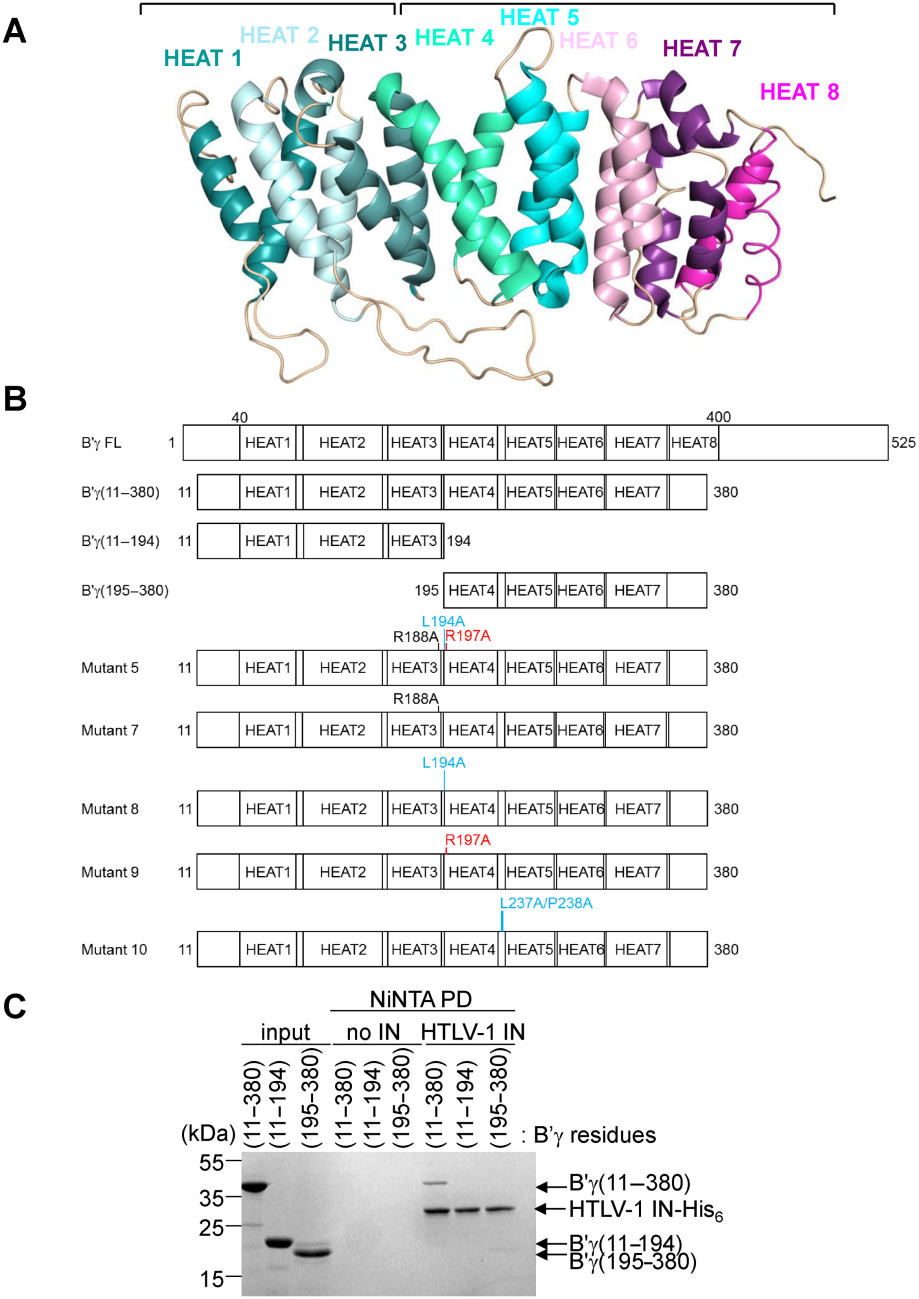
(**A**) Cartoon representation created in Pymol of B′γ1 (PDB 2IAE) (27) showing the pseudo-HEAT repeats in different colours, and labelled above the repeats as HEAT 1–8. The brackets on top of the cartoon refer to the deletion mutants designed to express HEAT repeats 1–3 (B′γ (11–194)) and 4–8 (B′γ(195–380). (**B**) Schematic representation of the pseudo-HEAT repeats in B′γ. The deletion mutants and point mutants used in this work are drawn underneath the full length (FL) protein. Start and end residues of the deletion fragments are indicated. Residue(s) indicated in red, blue and black are respectively critical, important and not involved in the interaction with HTLV-1 and -2 IN. (**C**) Ni-NTA pull-down done as described in Figure 3. Binding of B′γ(11–380), B′γ(11–194) and B′γ(195–380) to HTLV-1 IN-His_6_ was tested. Input and pull-downs are indicated above the gels. Migration of B′γ deletion fragments and HTLV-1 IN-His_6_ is indicated to the right of the gel. Migration of the molecular weight marker (in kDa) is indicated to the left of the gel. All gels were stained with Coomassie.

**Figure 7.**
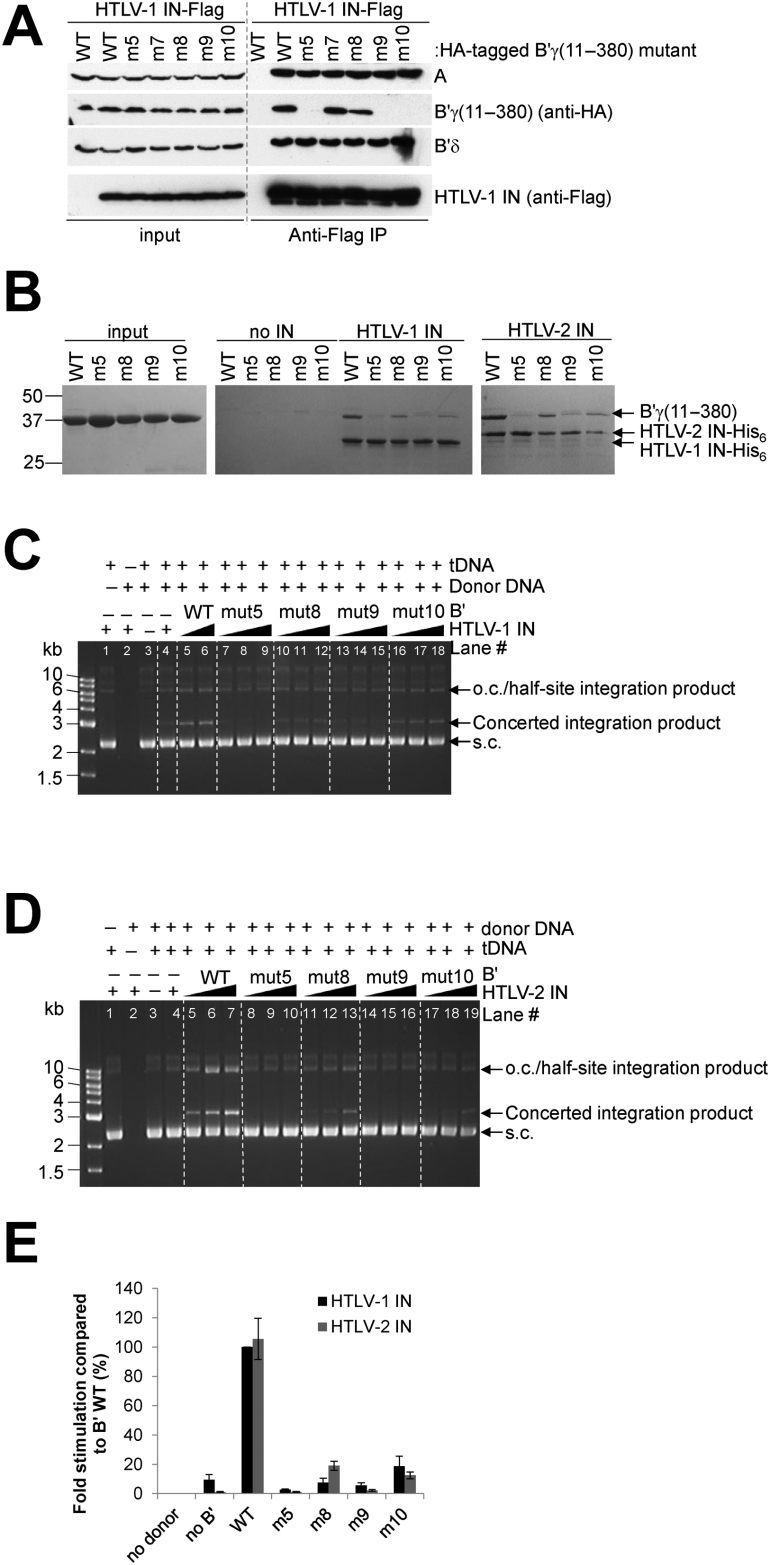
B′γ R197, L237 and P238 are critical for the interaction with HTLV-1 IN. (**A**) Flag-IPs with HTLV-1 IN-Flag and HA-tagged B′γ(11–380) wild type and point mutants as indicated above the gel. Antibodies used are indicated to the right of the blots. This is not a composite figure; the vertical line was added as a border between input and IP samples. (**B**) Coomassie stained gel of Ni-NTA pull-downs using HTLV-1 IN-His_6_ or HTLV-2 IN-His_6_ as bait, with wild type and B′γ(11–380) point mutants as indicated above the gel. (**C**–**E**) B′γ(11–380) point mutants unable to bind to HTLV IN do not stimulate concerted integration activity. (**C**) Reactions for HTLV-1 IN. All lanes, except lane 1 where donor was omitted, have donor DNA (pre-processed 20-mer). Lane 2: no tDNA, lane 3: no IN or B′γ(11–380), lane 4: no B′γ(11–380). Concentrations for HTLV-1 IN and B′γ(11–380) are as follows: 4 μM HTLV-1 IN with either 0.25 μM, 0.5 μM or 1 μM B′γ(11–380) WT or mutants was used in the reactions as indicated above the gel. (**D**) Reactions for HTLV-2 IN. All lanes, except lane 1 where donor was omitted, have donor DNA (pre-processed 24-mer). Lane 2: no tDNA, lane 3: no IN or B′γ(11–380). Lane 4; no B′γ(11–380), lanes 4–19 1 μM HTLV-2 IN. Lanes 5–7; 8–10; 11–13; 14–16; 17–19: respectively, 0.125 μM, 0.25 μM and 0.5 μM of WT, mutant 5, mutant 8, mutant 9 or mutant 10 B′γ(11–380) as indicated above the gels. Reactions were done for 1 h at 37°C. DNA products were separated on a 1.5% agarose gel and stained with GelRed. The 1 kb DNA ladder (NEB, indicated to the left of the gel) was used as a reference. (C) and (D) are not composite images; vertical lines were added in between the reactions done with different B′γ mutants to simplify the image. (**E**) Reactions were done as described above. Products of concerted integration were quantified as described previously (21) using the molar ratio of 2:1 IN : B′γ. Donor S20PQ and S24PQ were used for HTLV-1, respectively, HTLV-2 IN. A standard curve was made by serial dilution of the condition using WT B′γ. Fold stimulation is expressed in comparison to the reactions with WT B′γ, arbitrarily set to 100%. Averages and standard deviations are shown calculated on the data obtained from three independent experiments.

To further map the interaction of IN binding to B′γ, a set of deletion mutants in which pseudo-HEAT repeats were removed from the N- or C-terminal end were designed, expressed and purified from bacteria (Figure 6A and B). B′ fragments separated between pseudo-HEAT repeats 3 and 4 displayed reduced binding to HTLV-1 IN (Figure 6C). Following this serendipitous observation, a set of B′ mutants, expressed in HEK293T cells in HA-tagged form were tested for their ability to co-IP with HTLV-1 IN-Flag. Since δ-retroviral INs interact with the B′ PP2A holoenzyme (Figures 1 and 2A), only exposed residues surrounding pseudo-HEAT repeats 3 and 4 not involved in binding to the structural or catalytic subunit were chosen for mutagenesis (Figure 6B). B′γ(11–380)mutant 5, in which Arg188, Leu194 and Arg197 were mutated to Ala was unable to co-IP with Flag-tagged HTLV-1 IN (Figure 7A). To identify the residues critical for the interaction single point mutants, B′γ(11–380)mutant 7 (R188A), mutant 8 (L194A) and mutant 9 (R197A) were produced (Figure 6B). In addition, given the close proximity of the loop connecting pseudo-HEAT repeats 4 and 5, residues Leu237 and Pro238 were mutated to Ala in mutant 10 (Figure 6B). Whilst R188 does not appear to be involved in binding to HTLV-1 IN, L194 and more significantly R197 were critical for the interaction (Figure 7A). Mutant 10 was also unable to co-precipitate with Flag-tagged HTLV-1 IN (Figure 7A), whilst individually mutating residues L237 and P238 only partially reduced binding to HTLV-1 IN (data not shown). Interestingly, the scaffold subunit Aα co-precipitated with Flag-tagged HTLV-1 IN in extracts made from cells expressing mutant B′γ proteins incapable of binding to HTLV-1 IN. This is most likely due to the interaction of HTLV-1 IN with endogenous B′ isoforms. Indeed, endogenous B′δ co-precipitated with Flag-tagged HTLV-1 IN irrespective of which B′γ mutant was expressed (Figure 7A). Of importance, none of the mutations introduced into the B′γ protein affected its binding to the scaffold and catalytic subunits (Supplementary Figure S6) illustrating that these mutations do not alter the overall structure of the B′ proteins. This further corroborates the observation that residues L194, R197, L237 and P238 are involved in direct interaction with HTLV-1 IN.

To further investigate the importance of these interactions, mutants 5, 8, 9 and 10 were cloned, expressed and purified from bacteria and their binding to HTLV-1 and HTLV-2 IN was investigated using Ni-NTA pull-downs. As expected, mutants 5 and 9 were unable to associate with HTLV-1 and HTLV-2 IN-His_6_, whilst mutant 8 and 10 retained some residual binding to HTLV-1 and -2 IN (Figure 7B).

**Figure 8.**
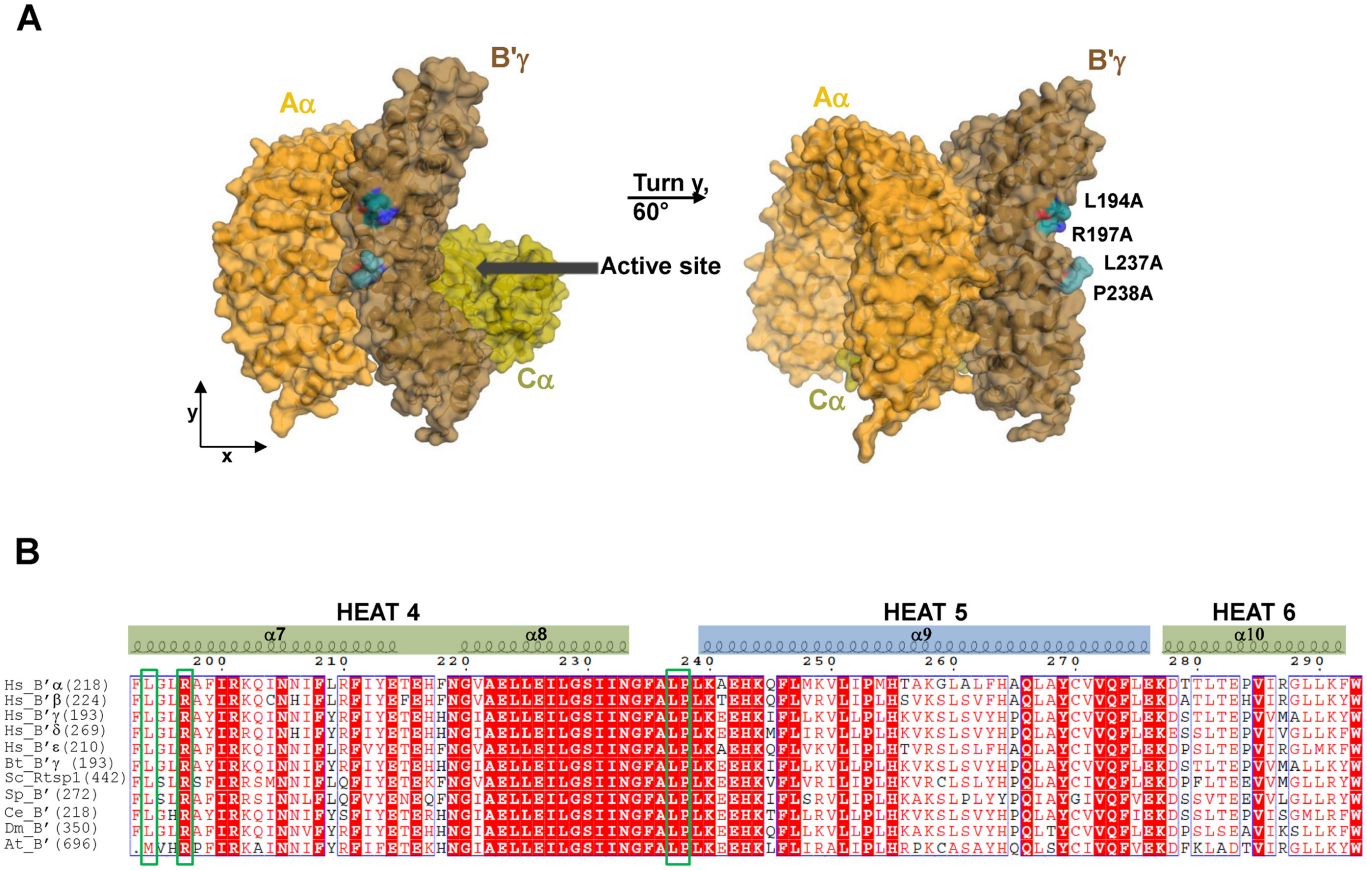
B′γ residues involved in binding to HTLV-1 IN are highly conserved and orient away from the substrate binding site. (**A**) Surface representation of the B′-PP2A holoenzyme based on PDB: 2IAE (27). The scaffold subunit (Aα) is shown in orange, the catalytic subunit (Cα) in yellow and the B′ regulatory subunit in brown. Residues involved in binding to HTLV-1 IN are shown as sticks and coloured as follows: carbon, green; oxygen, red; nitrogen, blue. The active site, at the interface between B′γ and Cα, is indicated. (**B**) Residues involved in binding to HTLV-1 and -2 IN (highlighted with green boxes) are highly conserved. Alignment of HEAT repeats 4 and 5 of human B′γ regulatory subunit (Hs_B′γ, GI: 31083259) with human B′α (Hs_B′α, GI: 5453950), B′β (Hs_B′β, GI: 5453952), B′δ (Hs_B′δ, GI: 5453954), B′ϵ (Hs_B′ϵ, GI: 5453956), bovine B′γ (*Bos taurus*, Bt_B′γ, GI: 134085946), and B′ isoforms from *Saccharomyces cerevisiae* (Sc_Rts1p, GI: 532526), *Schizosaccharomyc*es *pombe* (Sc_B′, GI: 19075706), *Caenorhabditis elegans* (Ce_B′, GI: 17557914), *Drosophila melanogaster* (Dm_B′, GI: 60677747) and *Arabidopsis thaliana* (At_B′, GI: 2244898). Numbers in brackets indicate the residue number of the respective isoform at which the alignment starts. Numbers on top of the alignment correspond to residue numbers in the human B′γ isoform. Secondary structures indicated are based on PDB: 2IAE (27). Alignments were made in Clustal Omega (53) (http://www.ebi.ac.uk/Tools/msa/clustalo/) and the figure was prepared using Espript 3.x (54) (http://espript.ibcp.fr/ESPript/ESPript/). For alignment with all bovine B′ regulatory subunits, see Supplementary Figure S7.

As expected, mutants 5 and 9 failed to stimulate concerted integration of HTLV-1 and -2 IN. In agreement with the observed reduction in binding affinity for HTLV-1 and -2 IN, point mutants 8 and 10 were also significantly (5- to 10-fold) less able to stimulate strand transfer activity of these δ-retroviral INs (Figure 7C–E). Of interest, B′γ residues L194, R197, L237 and P238 point away from the described PP2A substrate binding site (Figure 8A) and are conserved in all five human and bovine B′ isoforms (Figure 8B and Supplementary Figures S3 and S7), explaining the present interaction between bovine IN and human B′-PP2A (Figure 1 and Supplementary Table S2). Moreover, except for B′γ(L194) which is a Met in *Arabidopsis thaliana* B′, R197, L237 and P238 are conserved in *Saccharomyces cerevisiae*, *Schizosaccharomyces pombe*, *Caenorhabditis elegans*, *Drosophila melanogaster* and *Arabidopsis thaliana* B′ homologues (29) (Figure 8B).

## DISCUSSION

BLV and HTLV-1 IN bind specifically to the B′ PP2A holoenzyme. Whilst numerous peptides were identified for the scaffold and B′ regulatory subunits only few (BLV IN) or none (HTLV-1 IN) were identified for the catalytic subunit. This is most likely due to the fact that the catalytic subunit co-migrates with BLV and HTLV-1 IN in SDS-PAGE gels and the bands corresponding to the IN proteins were not analysed by tandem mass spectrometry. Nevertheless, independent Flag-IPs followed by western blot detection illustrates that both delta-retroviral INs specifically co-precipitate the PP2A holoenzyme containing B′ regulatory subunit (Figure 2A). PP2A is an essential and ubiquitous protein phosphatase that can make up 0.1–1% of the total cellular protein (32). A highly versatile enzyme, it plays critical functions in cellular homeostasis by being involved in a wide range of processes including DNA damage response, cell signalling, chromosome segregation, and apoptosis (18,19,33). The phosphatase is frequently inactivated in acute myeloid leukaemia (34). Its activity is modulated at multiple levels, and the specific roles of its diverse regulatory isoforms are only starting to emerge (35–41). Although δ-retroviral INs could be a substrate of B′-PP2A, phosphorylated forms of HTLV-1 or BLV IN were not detected in HEK293T cell extracts (data not shown). Moreover, recombinant protein used in this work was expressed and purified from *E. coli*, known to lack the kinase/phosphatase system present in eukaryotes. The direct interaction observed between recombinant B′ and HTLV-1 and -2 IN therefore suggests that phosphorylation is not critical for the interaction or the stimulation of integration activity by B′. Moreover, mapping of the B′ residues critical for the interaction with HTLV IN indicates that the interaction does not involve the canonical substrate binding interface of B′-PP2A (Figure 8A). However, 3 of the 4 identified residues involved in binding to δ-retroviral INs are conserved in eukaryotes, from yeast to humans (Figure 8B), suggesting that these residues could be involved in binding to other (conserved) endogenous protein(s) or substrate(s). Of importance, HTLV-1, -2 and BLV IN only share 38% sequence identity suggesting a strong selective pressure to preserve this virus-host interaction.

It is presently unclear what role B′-PP2A plays in δ-retroviral infection. It is likely that binding of HTLV-1 IN to B′-PP2A is beneficial to the virus for more than one reason. HTLV IN is not the first viral protein known to associate with PP2A. Simian virus 40 small T antigen and polyoma virus middle T antigen disrupt the PP2A holoenzyme to replace the regulatory subunit, hereby inhibiting PP2A activity which contributes to cellular transformation (18,19). Also the HTLV accessory protein Tax, a potent oncoprotein (42), was shown to bind to PP2A and thereby stimulate cell proliferation (43). Although Tax is only expressed in ∼40% of the ATL cases, it is thought to contribute to the early stages of cellular transformation (44). When investigating the effect of HTLV-1 or -2 IN on B′-PP2A activity, a 25–30% reduction in B′-PP2A activity could be observed only when the INs were present in a 5-fold molar excess to B′-PP2A. Thus, although B′ alone is sufficient to stimulate IN activity and IN does not influence B′-PP2A activity when present in a 2-fold molar excess of IN to B′-PP2A, it is too early to conclude that the functional interaction between IN and B′-PP2A is completely independent of B′-PP2A activity. Indeed, it is currently unknown what the local concentration of IN is when it meets B′-PP2A during infection. Moreover, it is at present impossible to disregard the possibility that binding of HTLV IN, or the HTLV PIC, to B′-PP2A influences its enzymatic activity towards, or its ability to associate with a certain substrate(s).

It will be important to determine if the interaction between HTLV-1 IN and B′-PP2A can influence cell cycle checkpoints and/or cellular transformation at the very early stages of infection and whether PP2A is inactivated in malignant ATL clones.

Following 3′ processing, the PIC needs to engage host chromatin to catalyse strand transfer. Lentiviruses and mouse mammary tumour viruses are unique in usurping the endogenous nuclear import machinery, whereas all other retroviruses depend on nuclear envelope breakdown during mitosis to bring the PIC into contact with chromatin (45,46). By its association and dephosphorylation of cohesin, B′-PP2A was shown to play a role in metaphase to anaphase progression (19) and could thus facilitate nuclear import of the HTLV PIC.

B′-PP2A dephosphorylates transcription factors such as p53, STAT1 and HDACs (47,33), of which cognate TFBs were found enriched close to HTLV-1 integration sites in a small but significant fraction of the infected cells (17). Unlike the best characterized retroviral IN host factors, LEDGF/p75 and Brd2–4, B′-PP2A does not directly associate with chromatin, but it can link the PIC to chromatin through any of these (and other yet unidentified) binding partners/substrates. Many of these factors also co-precipitated with HTLV-1 IN (Supplementary Table S3). More work is required to investigate the importance of these associations.

HTLV-1 is a notoriously difficult virus to work with; cell-to-cell transmission occurs through the virological synapse (48), and cell-free transductions using HLTV-derived vectors are ∼1000-fold less efficient than lentiviral transductions (49). Moreover, δ-retroviral INs associate with B′ of which there are at least five family members comprising minimally 10 (splice/translational) isoforms (19). Although it was shown that only the B′γ and B′δ isoforms are nuclear in a cell cycle dependent manner (35,50), all five B′ isoforms interact with centromeres/kinetochores during prometaphase (51). Thus, if (one of) the roles of B′-PP2A is to target the HTLV PIC to the site of integration, given the partial functional redundancy of the different B′ isoforms (52) and the fact that HTLV requires cells to go through mitosis to establish infection, any of these 10 isoforms could be involved. Of importance, whereas knock-down of individual B′ subunits does not adversely affect cell viability, targeting multiple B′ subunits is detrimental for cell survival (26). As a consequence, simple shRNA mediated approaches might not reveal whether B′-PP2A is critical for HTLV-1 infection and/or integration site selection. The use of PP2A inhibitors is an attractive alternative, although no distinction is made between B′ containing and other heterotrimeric PP2A holoenzymes using these chemical compounds (18).

## CONCLUSIONS

B′-PP2A is a functional δ-retroviral IN binding partner that displays some key characteristics of a PIC targeting factor: the interaction between B′-PP2A and IN is genus-specific, both IN and B′-PP2A co-localize in the nucleus of the cell, and B′ significantly stimulates concerted integration of δ-retroviral INs. Current investigations are focussed on determining whether PP2A targets the HTLV-1 PIC to its site of integration. It appears that HTLV INs when present in a 2-fold molar excess do not influence the activity of B′-PP2A on a phospho-Thr peptide. However, more work is needed to verify whether certain PP2A substrate(s) are affected by the association of B′-PP2A with this viral enzyme. Site directed mutagenesis carried out in this work has identified three highly conserved B′ residues critical for the interaction with and stimulation of HTLV-1 and -2 IN activity. Further characterization of the B′-PP2A : HTLV-1 IN interaction interface is ongoing with the goal of identifying the HTLV-1 IN residues involved in binding to B′. This information can then be used to further identify the role(s) of PP2A in δ-retroviral infection.

## SUPPLEMENTARY DATA

Supplementary Data are available at NAR Online.

SUPPLEMENTARY DATA
