# Supplementary material for: B′-protein phosphatase 2A is a functional binding partner of delta-retroviral integrase
Source: Nucleic Acids Res. 2015 Dec 10;44(1):364–76. doi: 10.1093/nar/gkv1347 (PMC4705670; doi:10.1093/nar/gkv1347)
Supplement: SUPPLEMENTARY DATA [file supp_44_1_364__index.html]

B′-protein phosphatase 2A is a functional binding partner of delta-retroviral integrase — B′-protein phosphatase 2A is a functional binding partner of delta-retroviral integrase — SUPPLEMENTARY DATA 

# B′-protein phosphatase 2A is a functional binding partner of delta-retroviral integrase

## SUPPLEMENTARY DATA

- SUPPLEMENTARY DATA
