# Supplementary material for: B′-protein phosphatase 2A is a functional binding partner of delta-retroviral integrase
Source: Nucleic Acids Res. 2015 Dec 10;44(1):364–76. doi: 10.1093/nar/gkv1347 (PMC4705670; doi:10.1093/nar/gkv1347)

SUPPLEMENTARY MATERIALS AND METHODS

DNA constructs for eukaryotic expression

A list with primer sequences can be found in Supplementary Table S4. The retroviral vectors used to express C-terminally Flag tagged HTLV-1, BLV, PFV, Feline Leukemia Virus (FeLV), Moloney Leukemia Virus (MLV), Mason Pfizer Monkey Virus (MPMV) IN, were described elsewhere (15). To make pQCt-Flag puro^R plasmid, pQCXIP (Clontech) was modified to contain the following sequence: *GCGGCCGC*-AGACACCATGGCG-*ACCGGT*-CAG-*CTCGAG*-GATTACAAGGATGACGATGACAAG-TGA-*GAATTC* (NotI-Kozak-AgeI-XhoI-Flag-Stop-EcoRI, where the restriction enzyme recognition sites are in italic). pQHTLV-1 IN^s/ΔNTD-Flag, expressing HTLV-1 IN residues 53-299, was made by amplifying the HTLV-1 IN^s/ΔNTD coding sequence using pQHTLV-1 IN^s-Flag (15) as a template and primers GM87 and GM88. The amplicon was digested with AgeI and XhoI and ligated into AgeI/XhoI digested pQCt-Flag-puro^R plasmid.

All constructs made expressing B' regulatory subunits were named with the gene name (PPP2R5C, D and E) whilst the proteins are labelled as B'γ, B'δ and B'ε. To express EGFP-B'γ(11-380), *PPP2R5C(11-380)* was PCR amplified with primers GM142 and GNM283 using the Stockholm clone PPP2R5CA-c005 (28) as a template, and cloned in between restriction sites EcoRI/SalI of pEGFP-C2.

pQHA-PPP2R5C(11-380) was cloned to express HA-tagged B'γ(11-380) in 293T cells. Hereto, *PPP2R5C(11-380)* was amplified using pEGFP-PPP2R5C(11-380) as a template with primers GNM336 and GNM337 and cloned in AgeI/EcoRI digested pQHA-puro^R (55). To clone the Flag-tagged version, the amplicon generated with primers GNM336 and GNM337 and digested with AgeI/EcoRI were ligated into linearized pQFlag-puro^R (55). Full-length B'γ was amplified using HeLa cDNA as a template with primers GNM367 and GNM380. Following AgeI/SalI digestion the amplicon was ligated into AgeI/XhoI digested pQFlag-puro^R. pQHA-PPP2R1A was generated by PCR amplifying the *PPP2R1A* open reading frame (ORF) using primers GM96 and GM97 and cloned into AgeI/XhoI digested pQHA-puro^R. pQMyc-puro^R was generated by ligating annealing primers GM70 and GM71 into MfeI/AgeI digested pQFlag-puro^R. To make pQMyc-PPP2CA, the *PPP2CA* ORF was amplified using primers GM98 and GM99 and pCDF-H6P-PPP2ACA (see below) as template and ligated into AgeI/BamHI digested pQMyc-puro^R. Point mutations were introduced by site directed mutagenesis. All plasmids were sequence verified.

DNA constructs for prokaryotic expression

The prokaryotic expression construct for His_6-TEV-B'γ(11-380) (PDB ID: 2JAK) (28) was obtained from Source Bioscience UK Limited and provided from the University of Oxford Structural Genetics Consortium (Stockholm clone name PPP2R5CA-c005). Constructs used to express C-terminally His_6-tagged HTLV-1 IN was described previously (56), and pHTLV-2 IN-His_6 was kindly provided by Peter Cherepanov (Cancer Research UK, Clare Hall Laboratories). Unless stated otherwise, all ORFs were amplified using a HeLa cDNA library (Clontech) as template. pCDF-H6P-PPP2CA was generated by amplifying the *PPP2CA* gene using primers GM93 and GM95 followed by amplification of this PCR product with primers GM94. This produces the following sequence His_6-BamHI-HRV 3C-EcoRI-PPP2CA-STOP-SalI-HindIII-NotI which allows the expression of an N-terminally His_6-fused protein of which the His_6-tag can be removed by Human Rhinovirus (HRV) 3C digestion. The *B"* ORF was amplified using primers GNM278 and GNM279 and cloned in between BamHI/SalI restriction site of pGEX-6P1 (GE Healthcare), giving pGM-GST-B". pCDF-H6P-PPP2R5C(11-194) and pCDF-H6P-PPP2R5C(195-380) were generated using the Stockholm clone PPP2R5CA-c005 (28) as a template with primers GM142 and GNM276 for deletion mutant B'γ(11-194) and GNM271 and GM143 for deletion mutant B'γ(195-380). The amplicons were digested with EcoRI/SalI (B'γ(11-194)) or EcoRI/NotI (B'γ(195-380)) and cloned in between the respective restriction sites of pCDF-H6P-PPP2CA. pET28a-SUMO-PPP2R5D(76-501) was amplified using GM112 and GM55 and ligated in between the BamHI/SalI restriction sites of pET28aSUMO (kindly given by Dr. Andre Ambrosio). For the expression of the α isoform of the scaffolding subunit, *PPP2R1A* was amplified using primers GM103 and GM97 and cloned in between the BamHI/XhoI restriction sites of pET28aSUMO. pCDF-H6P-PPP2R5E(51-401) was generated by ligating the amplicon made with primers GNM315 and GNM316 and digested with MfeI and SalI into EcoRI/SalI digested pCDF-H6P-PPP2CA. The HTLV-1 IN synthetic gene was amplified from pQHTLV-1 IN^S-Flag (15) using primers GM109 and GM110 and ligated into EcoRI/SalI digested pET28aSUMO, giving pET28aSUMO-HTLV-1 IN^s. The HTLV-2 IN ORF was amplified using pHTLV-2 IN-His_6 as a template with primers GM144 and GM131. This amplicon was digested with MfeI/SalI and ligated into EcoRI/SalI digested pET28aSUMO. All B'γ(11-380) point mutants described were sub-cloned into pET28a-SUMO to express as His_6-SUMO fusions to produce recombinant protein. All plasmids were sequence verified.

Protein purification

For expression of HTLV-1 IN-His_6, HTLV-2 IN-His_6, HIV-1 IN-His_6, FIV IN-His_6, and GST-B", the corresponding prokaryotic expression plasmids were transformed into the PC2 strain (22). Bacteria were grown in Luria Bertani medium at 30°C until an OD_{600nm} of 0.9 was

reached. The temperature was reduced to 25°C and protein expression was induced by addition of 0.01% IPTG. Four hours later, bacterial pellets were collected and stored at -80°C until use. All other recombinant proteins were expressed in the Rosetta2(DE3)pLacI strain (Novagen) and grown in Terrific Broth. Transformed bacteria grown in the appropriate selective media were allowed to reach an OD_{600nm} of 2.5-3 upon which the temperature was reduced to 25°C and protein expression induced by addition of 0.01% IPTG. Four hours later the bacteria were collected by centrifugation and pellets were frozen at -80°C until further use. All further procedures were done on ice or at 4°C. To purify the IN-His_6 proteins, bacterial pellets were thawed, resuspended and sonicated in core buffer (50 mM Tris pH7.4, 1 M NaCl, 7.5 mM CHAPS) supplemented with 1 mM PMSF. Cellular debris was removed by centrifugation at 50 000 *g*. Supernatant was supplemented with 10 mM imidazole and bound to HisSelect resin (Sigma). After extensive washes in wash buffer (core + 10 mM imidazole), IN proteins were eluted in 10 1 ml fractions with elution buffer (core buffer + 200 mM imidazole). Positive fractions were pooled, supplemented with 5 mM DTT, concentrated and supplemented with 10% glycerol final concentration, aliquoted and snap frozen in N_2(l). To produce untagged IN proteins, the His_6-SUMO-HTLV-1 and 2 IN proteins eluted from the HisSelect column were supplemented with 5 mM DTT and Ulp1 sumo-protease to remove the His_6-SUMO tag. Digestion was done overnight at 4°C. Cleaved protein was then diluted four fold in ice cold buffer A (25 mM Tris pH7.4, 7.5 mM CHAPS) before binding to an SP sepharose column (GE Healthcare) equilibrated with 25 mM Tris pH7.4, 250 mM NaCl, 7.5 mM CHAPS. After extensive washes, untagged HTLV-1 or 2 IN was eluted by applying a linear NaCl gradient. Positive fractions were pooled and further purified by size exclusion chromatography (HiLoad 16/60 SD200 column) in 25 mM Tris pH7.4, 1 M NaCl, 7.5 mM CHAPS. Positive fractions were pooled, supplemented with 5 mM DTT, concentrated and snap frozen in N_2(l). Purification of B'γ(11-380) was done as previously described (28). Purification of B'γ(11-194), B'γ(195-380), and B'ε(51-401) was done as for wild type B'γ(11-380) except that the His_6-tag was cleaved off by HRV 3C protease. The purification of His_6-SUMO-tagged B'γ(11-380) point mutants was slightly different from the His_6-tagged versions. Bacterial pellets were resuspended in 25 mM TrisHCl pH7.4, 0.5 M NaCl, 1 mM PMSF, supplemented with 0.1 mg/ml lysozyme, sonicated and the soluble fraction was bound to HisSelect. After extensive washes in the used sonication buffer supplemented with 10 mM imidazole, His_6-SUMO-B'γ(11-380) point mutants were eluted by increasing the imidazole concentration to 200 mM. Positive fractions were pooled, supplemented with 5 mM DTT and the His_6-SUMO tag was cleaved off by Ulp1 protease treatment overnight at 4°C. Following a 5 fold dilution of the cleaved protein in 25 mM Tris pH7.4, the proteins were purified as wild type B'γ(11-380). His_6-SUMO-Aα was expressed and purified as the His_6-SUMO-B'γ(11-380)

point mutants, with the exception that size exclusion chromatography of the untagged protein was done in ice cold 25 mM Tris pH7.4, 500 mM NaCl. B'δ(76-501) was expressed as a His_6-SUMO fusion protein. The bacterial pellets were resuspended in 25 mM Tris pH7.4, 150 mM NaCl, 1 mM PMSF. After sonication, the soluble supernatant was bound to HisSelect, after elution the His_6-SUMO-tag was removed by Ulp1 cleavage overnight. The untagged protein was then purified by size exclusion in 25 mM Tris pH7.4, 150 mM NaCl. Positive fractions were pooled, supplemented with 5 mM DTT, concentrated and flash frozen in N_2(l). GST-B'' was extracted from the bacterial pellets in 25 mM Tris pH8, 100 mM NaCl, 1% TX-100, 1 mM $CaCl_2$, 1 mM PMSF. Following sonication and removal of debris by centrifugation, GST-B'' was allowed to bind glutathione sepharose (GE Healthcare). After extensive washes B'' was released from the beads by HRV 3C protease digestion overnight. Untagged B'' was further purified by anion exchange chromatography (linear NaCl gradient from 50 mM to 500 mM). Positive fractions were pooled, supplemented with 2 mM DTT, concentrated and flash frozen in N_2(l).

Tissue culture, stable cell lines and immunostaining

HEK293T and HeLa cell lines were maintained in Dulbecco's Modified Eagle Medium (Sigma) supplemented with 10% fetal bovine serum (Sigma), 100 IU/mL penicillin, and 100 μg/mL streptomycin (Sigma). The HEK293T cell line stably expressing Flag-tagged HIV-1 IN^s was published previously (57), and was maintained in 300 μg/ml hygromycin B supplemented medium. Retroviral particles were produced as described previously (55). Fourty eight h post-infection the cells were selected with 0.5 μg/ml puromycin.

For immunostaining, HeLa cells were plated out in 8-well Lab-Tek II Chamber slides (Nunc) to reach 80% confluence the next day. HeLa cells were transfected using 150 ng of plasmid DNA in total by X-tremeGENE 9 transfection reagent (Roche) following manufacturer's instructions. Twenty h post-transfection the cells were fixed for 10 min in 4% paraformaldehyde (diluted in phosphate buffered saline (PBS)) followed by permeabilization using 0.1% Triton X-100 diluted in PBS. All antibodies were diluted in blocking buffer (10% FBS, 20 mM NH_4Cl in PBS). Flag-tagged IN proteins were detected using the monoclonal M2 anti-Flag antibody (Sigma, 1:500), and EGFP-B'γ(11-380) was detected using the rabbit anti-EGFP antibody (Life Technologies, 1:2000). Goat anti-mouse IgG conjugated to Texas Red (Life Technologies) and Alexa 488 conjugated goat anti-rabbit IgG (Life Technologies) were diluted 1:400. DNA was visualized by 4',6-diamidino-2-phenylindole (DAPI, Life Technologies) staining. Images were acquired using an Olympus microscope with a 60x Plapon oil objective (NA 1.4). DAPI was excited with a 405 nm laser beam, whilst 488 nm,

respectively 559 nm laser beams were used to excite the Alexa 488 and TexasRed dyes. Images were acquired sequentially at 40 μs/pixel, 1024x1024 image resolution.

Immunoprecipitation (IP) and Western blot

Large scale IPs were done on extracts made from cells grown to ~80% confluence on 8 500 cm^2 dishes. Cells were harvested by trypsinization, washed in ice cold PBS and resuspended in 5 pellet volumes of freeze-thaw buffer (FTB, 10 mM Hepes pH7.1, 10 mM KCl, 1.5 mM $MgCl_2$, 0.34 M sucrose, 150 mM NaCl, 10% glycerol, 1 mM DTT, 1 mM PMSF) supplemented with Complete EDTA free (Roche). Resuspended cells were frozen at -80°C. To make the extracts, the resuspensions were allowed to thaw fast at 37°C and immediately centrifuged at 16 000 *g*, 30 min at 4°C. Supernatants were collected, supplemented with 0.1 M NaCl, 0.5 % Nonidet P-40 (NP-40) and 0.5 mM PMSF. The extracts were pre-cleared over 100 μl washed Protein G agarose (GE Healthcare) followed by binding to 100 μl anti-Flag agarose (Sigma). Flag-tagged protein complexes were allowed to bind to the beads for 3h by end-over-end rocking at 4°C. Beads were washed extensively with wash buffer (FTB supplemented with 0.5 % NP-40, 0.1 M NaCl) and bound proteins were eluted with 0.04 mg/ml Flag peptide (Sigma) in wash buffer. Eluted proteins were precipitated by trichloric acid, pellets were dissolved in SDS loading buffer and proteins were separated on a 4-20% BisTris gel (Life Technologies). The gels were stained in Colloidal Coomassie (Sigma) and bands were excised and sent for tandem mass spectrometry analysis to the Taplin Mass Spectrometry Facility. The MS data was analyzed by the Taplin Mass Spectrometry facility and Sequest was used to search data. The data was filtered based on XCorr and dCn values and then manually inspected for proteins that only had three or few peptide matches. Values of 1.5 for peptides with one or two charges, 3.0 for three charges for XCorr and 0.1 or higher for dCn were used. The data was also searched allowing for either partial tryptic peptides or with no enzyme specificity and required that all peptides be tryptic. Only proteins with minimally 2 unique peptide matches, and that were absent in the negative control sample are listed in Supplementary Tables S2 and S3.

For small scale IPs, 293T cells grown in 1 10 cm dish, were harvested by trypsinization and washed in ice cold PBS. All procedures were done on ice or at 4°C. Cells were lysed in 5 volumes of IP buffer (10 mM TrisHCl pH7.5, 150 mM NaCl, 10% glycerol, 1% NP-40, 2 mM $MgCl_2$, Complete EDTA free (Roche), 2 mM DTT), left on ice for 10 min and cellular debris was removed by centrifugation at 16 000 *g* for 30 min. To verify the binding between Flag-tagged B'γ(11-380) and the scaffold and catalytic subunit, IP buffer without detergent was used. Supernatants were allowed to bind to 25 μl pre-washed anti-Flag agarose beads (Sigma) by end-over-end rocking at 4°C. Beads were washed 4 times in 1 ml of IP buffer.

After removing all remaining liquid from the beads, proteins were eluted by boiling the beads in 45 μl of Laemmli buffer. After separation of the proteins on an 11% SDS-PAGE denaturing gel, proteins were electrotransferred onto nitrocellulose membrane. Blots were blocked in 5% milk/PBS and probed with the following antibodies: horse radish peroxidase (HRP)-conjugated mouse anti-Flag (Sigma, 1:2000), rabbit anti-HA (Origin, 1:500), rabbit anti-B'δ (Bethyl Laboratories, 1:500), rabbit anti-Aα (Bethyl Laboratories, 1:2000), rabbit anti-B (GeneTex, 1:500), mouse anti-Cα (Becton Dickinson, 1:500), rabbit anti-STRIATIN4 (Bethyl Laboratories, 1:1000), rabbit anti-Myc (Sigma, 1:2000). All antibodies were diluted in 5% milk/PBST (PBS supplemented with 0.1% Tween-20). HRP conjugated goat anti-rabbit and sheep anti-mouse antibodies were used at 1:2000 dilution (GEHealthcare). The blots were developed using Clarity ECL (BioRad). Blots shown are representative of 2-4 independent experiments.

Phosphatase assays

To isolate B'-PP2A holo-enzymes from mammalian cells, a HEK293T cell line was generated that stably expresses full-length wild type Flag-B'γ by retroviral transduction as described above. The cell line was maintained in 0.5 μg/ml puromycin. Flag-B'γ-PP2A holo-enzymes were purified as described previously (31) and verified by gel and western blot to confirm the presence of all three subunits. To quantify the amount of Flag-B'γ-PP2A purified, 10 μl was separated on an 11% SDS-PAGE gel next to a dilution series of BSA. Following silver staining, using ImageJ it was estimated that the concentration of holo-enzyme in our eluate was 28 nM. The colorimetric malachite green phosphatase assay was used to measure PP2A enzymatic activity using the PP2A specific phospho-Threonine peptide (K-R-pT-I-R-R) as a substrate. Absorbance was read at 620nm. A standard curve was made using a dilution series of potassium phosphate ranging from 0 to 2000 pmoles phosphate. Reactions with the phospho-Thr substrate were done in the following phosphatase assay buffer: 25 mM Tris-HCl pH 7.4, 1 mM EDTA, 1 mM EGTA, 1 mM DTT and 0.25 mg/ml BSA (31) and allowed to take place for 30 min at 37°C before the malachite green substrate was added. Absorbance was measured following a 15 min incubation at room temperature with the colorimetric substrate. Phospho-Thr peptide was used in large excess: 200 μM substrate vs 0.125 nM Flag-B'γ-PP2A holo-enzyme which results in a release of 1719±50 pmoles phosphate. The following controls were used: no peptide, no Flag-B'γ-PP2A, and pre-incubation of Flag-B'γ-PP2A with 10 nM Okadeic acid. HTLV-1 and 2 IN were added to the reaction at 1:2, 1:1, 2:1 or 5:1 molar ratio of IN: Flag-B'γ-PP2A. An additional control where IN dilution buffer (25 mM Tris-HCl pH7.4, 500 mM NaCl, 2 mM DTT, 0.5% CHAPS) was added instead of IN was used to rule out interference of the buffer. The pmoles released

phosphate were calculated using the standard curve and % activity was calculated compared to the positive control reaction (200 μM phopho-Thr peptide substrate with 0.125 nM Flag-B'γ-PP2A). All reactions were done in triplicate.

Ni-NTA pull-downs

Five μg His_6-tagged bait protein was allowed to bind to 5 μg prey protein in a volume of 0.8 ml pull-down buffer (PDB, 25 mM TrisHCl pH 7.4, 150 mM NaCl, 2 mM DTT, 20 mM imidazole, 0.5% CHAPS), to which 40 μl of Ni-NTA slurry pre-equilibrated in PDB was added. Ten μg of BSA was added to reduce non-specific binding. After 3h of end-over-end rocking at 4°C, the Ni-NTA beads were pelleted by centrifugation (1 000 *g*, 2 min 4°C) and washed extensively in PDB. Bound proteins were eluted by boiling the beads in 20 μl 2x Laemmli buffer supplemented with 5 mM EDTA. Ten μl was loaded on gel. Representative gels of pull-downs repeated at least 3 times are shown.

SUPPLEMENTARY FIGURE LEGENDS

Supplementary Table S1: Sequences donor DNA substrates.

Supplementary Table S2: Proteins that co-immunoprecipitated with Flag-tagged BLV IN. Proteins of which 2 or more unique peptides were detected that specifically co-immunoprecipitated with Flag-tagged BLV IN.

Supplementary Table S3: Proteins that co-immunoprecipitated with Flag-tagged HTLV-1 IN. Proteins of which 2 or more unique peptides were detected that specifically co-immunoprecipitated with Flag-tagged HTLV-1 IN.

Supplementary Table S4: List of primers. Primer name and sequences used to generate the plasmids described in this manuscript.

Supplementary Figure S1. Binding between HTLV-1 IN and B'-PP2A resists high NaCl concentration. (A) Flag-IPs using the HEK293T cell line that stably expresses HTLV-1 IN-Flag, and the parental HEK293T cell line as a control. IPs were done with increasing NaCl concentration (150 mM, 250 mM and 450 mM) as indicated on top of the blot. This is not a composite image; the vertical line was added to distinguish the input from the IP samples. Antibodies used are indicated to the right of the blot.

Supplementary Figure S2. HTLV-1 but not PFV IN co-localizes with PP2A. HeLa cells were transfected to express either HTLV-1 IN -Flag or PFV IN-Flag together with EGFP-B'γ(11-380). Cells were fixed 20 hrs post-transfection and proteins were detected with anti-Flag and anti-GFP antibodies. DNA was stained using DAPI. Representative images of three independent experiments at different magnifications are shown. HTLV-1 IN and EGFP-B'γ(11-380) co-localized in ~97% of the cases (> 100 cells counted). Bars in the upper left hand corner of the panels represent 10 μm.

Supplementary Figure S3. Alignment of human B' regulatory isoforms. Shown are B'γ (GI: 31083259), B'α (GI: 5453950), B'β (GI: 5453952), B'δ (GI: 5453954), B'ε (GI: 5453956). Secondary structures indicated are based on PDB: 2IAE (28). Alignments were made in Clustal Omega (53) (http://www.ebi.ac.uk/Tools/msa/clustalo/) and the figure prepared using Espript 3.x (54) (http://espript.ibcp.fr/ESPript/ESPript/). Residues involved in binding to δ-retroviral INs are highlighted with a green box.

Supplementary Figure S4. B'γ(11-380) stimulates HTLV-2 IN concerted integration activity. Integration reactions done with HTLV-2 IN using either blunt (B) or pre-processed (P) 30-mer donor DNA as substrate. Assays done as described in Figure 5B. All reactions (except for lane 3 where no IN was added) contained 4 μM HTLV-2 IN and 2 μM B'γ(11-380). Lane 1: no donor, lane 2: no tDNA; lane 3: no IN; lane 4: blunt donor DNA 30-mer; lane 5: pre-processed 30-mer. Reactions were done for 90 min at 37°C. DNA products were separated on a 1.5% agarose gel and stained with GelRed.

Supplementary Figure S5. Up to twofold molar excess of HTLV-1 and -2 IN does not influence PP2A enzymatic activity on a phospho-Thr peptide. (A) Flag-B'γ-PP2A was purified from HEK293T cells and 10 μl was separated on gel followed by silver staining. A dilution series of BSA (as indicated above the gel) was used to quantify the amount of enzyme purified. (B) Three μl of the purified Flag-B'γ-PP2A was loaded on gel and probed with the antibodies indicated to the right of the blots. (C) PP2A phosphatase activity was measured as described in Supplementary Materials and Methods. % activity compared to the reaction without addition of IN is plotted. All reactions (except for controls, see below) contained the following: 200 μM phopho-Thr peptide, 0.125 nM Flag-B'γ-PP2A in phosphatase assay buffer. Reactions were allowed to take place for 30 min at 37°C before addition of colorimetric substrate. Negative controls: - peptide (no peptide); - PP2A (no enzyme control). + OA is the reaction where Flag-B'γ-PP2A was pre-treated with 10 nM Okadeic acid; + IN buffer, here the IN dilution buffer was added to the reaction. 1:2; 1:1; 2:1; 5:1 denotes the

molar ratio of IN to Flag-B'γ-PP2A. Results and standard deviations of three independent experiments are shown.

Supplementary Figure S6. B'γ point mutants unable to bind to HTLV IN associate with scaffold and catalytic subunit. Flag-tag (negative control) and Flag-tagged B'γ fusions (indicated above the gel) were transiently expressed together with HA-tagged Aα and Myc-tagged Cα. Input and Flag-IPs are indicated above the gel. Proteins detected by western blot are indicated to the right of the gel.

Supplementary Figure S7. Alignment of human B'γ (Hs_B'γ) regulatory subunit with bovine B' regulatory subunits (Bt_B'α-ε). Shown are *Bos taurus* B'α (GI: 126165212), Bt_B'β (GI: 115497700), Bt_B'γ (GI: 134085946), Bt_B'δ (GI: 329744636) and Bt_B'ε (GI: 134085749). Residues involved in binding to δ-retroviral INs are conserved and are highlighted with a green box. Secondary structures indicated are based on PDB: 2IAE (28). Alignments were made in Clustal Omega (53) (http://www.ebi.ac.uk/Tools/msa/clustalo/) and the figure prepared using Espript 3.x (54) (http://espript.ibcp.fr/ESPript/ESPript/).

ADDITIONAL REFERENCES

55. Maertens, G.N., El Messaoudi-Aubert, S., Elderkin, S., Hiom, K. and Peters, G. (2010) Ubiquitin-specific proteases 7 and 11 modulate Polycomb regulation of the INK4A tumour suppressor. *EMBO J*, **29**, 2553-2565.

56. Wang, T., Piefer, A.J. and Jonsson, C.B. (2001) Interactions of the human T-cell leukemia virus type-II integrase with the conserved CA in the retroviral long terminal repeat end. *J Biol Chem*, **276**, 14710-14717.

57. Cherepanov, P., Maertens, G., Proost, P., Devreese, B., Van Beeumen, J., Engelborghs, Y., De Clercq, E. and Debyser, Z. (2003) HIV-1 integrase forms stable tetramers and associates with LEDGF/p75 protein in human cells. *J Biol Chem*, **278**, 372-381.

Supplementary Table S1: Sequences donor DNA substrates

HTLV-1 donor DNA (Balakrishnan J Virol 1997)

Primer name	sequence (5'-)
S20UP	AGAGAAATTTAGTACACA
S20B	ACTGTGTACTAAATTTCTCT
S20UN	AGAGAAATTTAGTACACAAT
S20UPQ	GACTCACTATAGGGCACGCGTAGAGAAATTTAGTACACA
S20BQ	ACTGTGTACTAAATTTCTCTACGCGTGCCCTATAGTGAGTC

HTLV-2 donor DNA

Primer name	sequence (5'-)
S24UN	GTCTTCCCGGGGAAGACAAACAAT
S24B	ATTGTTTGTCTTCCCCGGGAAGAC
S24UP	GTCTTCCCGGGGAAGACAAACA
S30UN	AGCATTGTCTTCCCGGGGAAGACAAACAAT
S30B	ATTGTTTGTCTTCCCCGGGAAGACAATGCT
S30UP	AGCATTGTCTTCCCGGGGAAGACAAACA
S19UN	CCCGGGGAAGACAAACAAT
S19B	ATTGTTTGTCTTCCCCGGG
S19UP	CCCGGGGAAGACAAACA
S24UPQ	ATTGTTTGTCTTCCCCGGGAAGACACGCGTGCCCTATAGTGAGTC
S24BQ	GACTCACTATAGGGCACGCGTGTCTTCCCGGGGAAGACAAACA

Supplementary Table S2: Proteins that co-immunoprecipitated with Flag-tagged BLV IN

Identified PP2A subunits	# unique peptides
PPP2R1A (structural subunit, alpha isoform)	74
PPP2R1B (structural subunit, beta isoform)	36
PPP2R5A (regulatory subunit, B' alpha isoform)	29
PPP2R5C (regulatory subunit, B' gamma isoform)	58
PPP2R5D (regulatory subunit, B' delta isoform)	49
PPP2R5E (regulatory subunit, B' epsilon isoform)	34
PPP2CA (catalytic subunit, alpha isoform)	8

BLV IN co-precipitating proteins ordered according number of unique peptides	# unique peptides
DNAPKc 1	112
UBR5	27
RUVBL2/TIP48	23
DNAJA2	21
T complex protein 1 subunit 7	21
T complex protein 1 subunit 8	19
TCP1	18
CCT6A	15
DNAJA1	15
ILK-2	11
RUVBL1/TIP49	11
Isoform 1 of Thyroid receptor-interacting protein 13	7
Isoform 1 of HEC1/NDC80-interacting centrosome-associated protein 1	7
HAUS8, Isoform 1 of HEC1/NDC80-interacting centrosome-associated protein 1	7
Isoform ATE1-1 of Arginyl-tRNA-protein transferase 1	6
RCL2, reticulocalbin 2	6
26S proteasome non-ATPase regulatory subunit 3	5
weakly similar to Uro-adherence factor A (Fragment)	5
isoform 1 of CDC42 effector protein 1	5
T complex protein 1 subunit 3	4

Supplementary Table S3: Proteins that co-immunoprecipitated with Flag-tagged HTLV-1 IN

**Identified PP2A subunits**	**HTLV-1 IN # unique pept**
PPP2R1A (structural subunit, alpha isoform)	23
PPP2R5A (regulatory subunit, B' alpha isoform)	8
PPP2R5C (regulatory subunit, B' gamma isoform)	8
PPP2R5D (regulatory subunit, B' delta isoform)	17
PPP2R5E (regulatory subunit, B' epsilon isoform)	7

**HTLV-1 IN co-precipitating proteins ordered according number of unique peptides**	**HTLV-1 IN # unique pept**
Fanconi anemia group I protein	31
Huntingtin	22
Dynactin subunit 1	21
DNA excision repair protein ERCC-6-like	20
Insulin receptor substrate 4	19
Nuclear pore complex protein Nup93	19
CLIP-associating protein 1	18
RuvB-like 2	17
Structural maintenance of chromosomes protein 2	16
PAS domain-containing serine/threonine-protein kinase	15
Sperm-associated antigen 5	15
Rab3 GTPase-activating protein non-catalytic subunit	15
Leucine-rich PPR motif-containing protein, mitochondrial	15
CLIP-associating protein 2	14
Breakpoint cluster region protein	14
OTU domain-containing protein 4	14
Protein diaphanous homolog 1	13
Protein furry homolog-like	13
cAMP-dependent protein kinase type I-alpha regulatory subunit	12
Leucine-rich repeat-containing protein 40	12
Ataxin-10	12
Peroxisome biogenesis factor 1	12

Liprin-alpha-3	12
Zinc finger ZZ-type and EF-hand domain-containing protein 1	12
DNA-directed RNA polymerase III subunit RPC1	11
Thyroid receptor-interacting protein 13	11
Conserved oligomeric Golgi complex subunit 4	11
T-complex protein 1 subunit delta	11
Vacuolar protein sorting-associated protein 13A	11
Fanconi anemia group D2 protein	10
E3 ubiquitin-protein ligase HUWE1	10
DNA-dependent protein kinase catalytic subunit	10
Structural maintenance of chromosomes protein 1A	10
cDNA FLJ60082, weakly similar to Uro-adherence factor A (Fragment)	10
Serine-protein kinase ATM	10
Protein FAM83H	10
Liprin-alpha-1	9
TBC1 domain family member 9B	9
Elongator complex protein 1	9
T-complex protein 1 subunit alpha	9
Telomere length regulation protein TEL2 homolog	9
CARMIL1a	9
Calmodulin-regulated spectrin-associated protein 3	9
Splicing factor 3B subunit 1	9
Structural maintenance of chromosomes protein 3	9
Probable ATP-dependent RNA helicase YTHDC2	9
Pleckstrin homology domain-containing family A member 5	9
Protein timeless homolog	9
Pleckstrin homology-like domain family B member 2	8
Nuclear pore complex protein Nup160	8
Probable ATP-dependent RNA helicase DDX17	8
WD repeat-containing protein 19	8
Uridine 5'-monophosphate synthase	8
Phosphoribosylformylglycinamidine synthase	8

Isoleucyl-tRNA synthetase, cytoplasmic	8
TBC1 domain family member 15	8
T-complex protein 1 subunit gamma	8
Centromere/kinetochore protein zw10 homolog	8
Vimentin	8
DNA repair protein RAD50	8
Nephrocystin-3	8
Nuclear pore complex protein Nup155	8
Melanoma-associated antigen	7
TBC1 domain family member 4	7
HAUS augmin-like complex subunit 5	7
Late secretory pathway protein AVL9	7
Wings apart-like protein homolog	7
X-ray repair cross-complementing protein 6	7
T-complex protein 1 subunit theta	7
Fanconi anemia group A protein	7
Serine/threonine-protein kinase Nek1	7
Kinesin-like protein KIF7	7
Symplekin	7
Synergin gamma	7
Histone acetyltransferase type B catalytic subunit	7
Transducin-like enhancer protein 3	7
Cleavage and polyadenylation specificity factor subunit 1	7
26S proteasome non-ATPase regulatory subunit 3	7
DmX-like protein 2	7
Glucocorticoid receptor	7
Signal transducer and activator of transcription 3	7
Lipopolysaccharide-responsive and beige-like anchor protein	7
Intraflagellar transport protein 74 homolog	7
FK506-binding protein 15	7
Protein flightless-1 homolog	7
26S protease regulatory subunit 7	7

ATP-dependent RNA helicase A	7
DmX-like protein 1	7
Protein aurora borealis	6
Activating molecule in BECN1-regulated autophagy protein 1	6
RNA polymerase II-associated protein 1	6
SCL-interrupting locus protein	6
Beta/gamma crystallin domain-containing protein 3	6
Cullin-4B	6
HAUS augmin-like complex subunit 3	6
Sec1 family domain-containing protein 1	6
Clathrin interactor 1	6
Protein disulfide-isomerase A6	6
Negative elongation factor C/D	6
Tetratricopeptide repeat protein 27	6
FLJ00246 protein (Fragment)	6
Spliceosome RNA helicase BAT1	6
Vigilin	6
M-phase phosphoprotein 9	6
Brain-specific angiogenesis inhibitor 1-associated protein 2-like protein 1	6
Protein strawberry notch homolog 1	6
Replication factor C subunit 1	6
Peroxisomal targeting signal 1 receptor	6
Inverted formin-2	6
Conserved oligomeric Golgi complex subunit 5	6
Protein KIAA1731	6
Condensin complex subunit 1 (NCAPD2)	6
HEAT repeat-containing protein 2	6
Large proline-rich protein BAG6	6
Tetratricopeptide repeat protein 4	6
D-3-phosphoglycerate dehydrogenase	5
SCY1-like protein 2	5
Heterogeneous nuclear ribonucleoprotein M	5

Protein FAM83D	5
Arginyl-tRNA synthetase, cytoplasmic	5
RNA-binding protein 39	5
Probable helicase senataxin	5
Angiomotin	5
Prolyl 4-hydroxylase subunit alpha-1	5
Cytosolic carboxypeptidase 1	5
Coatomer subunit delta	5
Coatomer subunit alpha	5
Phosphoinositide 3-kinase regulatory subunit 4	5
Mitotic spindle assembly checkpoint protein MAD1	5
Serine/threonine-protein kinase	5
Nuclear-interacting partner of ALK	5
Importin subunit alpha-7	5
A-kinase anchor protein 8	5
Ribonuclease inhibitor	5
ADP-ribosylation factor GTPase-activating protein 2	5
ADP-ribosylation factor GTPase-activating protein 3	5
E3 ubiquitin-protein ligase TRIM33	5
ATP-dependent RNA helicase DDX1	5
Putative uncharacterized protein DKFZp459F246	5
Tight junction protein ZO-2	5
Protein FAM65A	5
Erythroid differentiation-related factor 1	5
Translational activator GCN1	5
E3 ubiquitin-protein ligase RNF14	5
Sister chromatid cohesion protein PDS5 homolog A (SCC-112)	4
Serine/threonine-protein kinase RIO2	4
Serologically defined colon cancer antigen 3	4
CTP synthase 1	4
Centrosomal protein of 170 kDa	4
A-kinase anchor protein 8-like	4

Nuclease-sensitive element-binding protein 1	4
Lamin-B1	4
cAMP-dependent protein kinase type II-alpha regulatory subunit	4
Low-density lipoprotein receptor-related protein 2	4
Probable ubiquitin carboxyl-terminal hydrolase FAF-X	4
Fanconi anemia group J protein	4
Protein LAS1 homolog	4
Protein disulfide-isomerase	4
Dual specificity mitogen-activated protein kinase kinase 7	4
26S proteasome non-ATPase regulatory subunit 4	4
Eukaryotic initiation factor 4A-III	4
RuvB-like 1	4
Nuclear pore complex protein Nup85	4
Myosin-IXa	4
DNA-directed RNA polymerase III subunit RPC5	4
Serine/threonine-protein kinase MST4	4
Centrosomal protein of 192 kDa	4
G2/mitotic-specific cyclin-B1	4
26S protease regulatory subunit 8	4
E3 ubiquitin-protein ligase RNF123	4
U4/U6 small nuclear ribonucleoprotein Prp31	4
Mitogen-activated protein kinase 6	4
Protein Hook homolog 1	4
Signal recognition particle 68 kDa protein	4
26S proteasome non-ATPase regulatory subunit 12	4
Sorting nexin-5	4
Protein FAM123B	4
WD repeat-containing protein 62	4
Deubiquitinating protein VCIP135	4
Tumor susceptibility gene 101 protein	4
Receptor-interacting serine/threonine-protein kinase 4	4
RNA-binding protein 14	4

Eukaryotic translation initiation factor 3 subunit E	4
Protein fat-free homolog	4
Glutaminyl-tRNA synthetase	4
Striatin-4	4
Protein Hook homolog 3	4
Protein Njmu-R1	4
Probable ATP-dependent RNA helicase DDX41	4
AP-3 complex subunit delta-1	4
Anaphase-promoting complex subunit 4	4
Baculoviral IAP repeat-containing protein 6	4
Conserved oligomeric Golgi complex subunit 6	4
La-related protein 1	4
Bardet-Biedl syndrome 1 protein	4
Cyclin-G-associated kinase	4
Dual specificity tyrosine-phosphorylation-regulated kinase 1A	4
Ubquitin/ribosomal protein S27Ae fusion protein (Fragment)	3
Melanoma-associated antigen D1	3
TRAF-type zinc finger domain-containing protein 1	3
General transcription factor II-I	3
Cytoskeleton-associated protein 2	3
CAD protein OS=Homo sapiens	3
Ubiquitin-associated protein 2-like	3
RAF proto-oncogene serine/threonine-protein kinase	3
A-kinase anchor protein 10, mitochondrial	3
Maternal embryonic leucine zipper kinase	3
Cellular tumor antigen p53	3
GTPase-activating protein and VPS9 domain-containing protein 1	3
Thyroid hormone receptor-associated protein 3	3
Actin nucleation promoting factor (Fragment)	3
Dynactin subunit 2	3
Uncharacterized protein C14orf80	3
Dual specificity mitogen-activated protein kinase kinase 2	3

mRNA cap guanine-N7 methyltransferase	3
Neurobeachin	3
Latent-transforming growth factor beta-binding protein 1	3
T-complex protein 1 subunit beta	3
Catenin beta-1	3
Niban-like protein 1	3
GSK3A protein	3
Synembryn-A	3
DDB1- and CUL4-associated factor 8	3
Tetratricopeptide repeat protein 31	3
Highly divergent homeobox	3
Coatomer subunit gamma-2	3
Spindle and kinetochore-associated protein 3	3
Mediator of RNA polymerase II transcription subunit 23	3
Transducin-like enhancer protein 4	3
Importin subunit alpha-1	3
Nuclear prelamin A recognition factor	3
Melanoma-associated antigen D4	3
Tetratricopeptide repeat protein 26	3
Transducin beta-like protein 3	3
Regulatory-associated protein of mTOR	3
TBC1 domain family member 9	3
Alstrom syndrome protein 1	3
ATP-dependent DNA helicase Q4	3
Trifunctional enzyme subunit beta, mitochondrial	3
CCR4-NOT transcription complex subunit 10	3
cDNA FLJ53892, highly similar to Pantothenate kinase 4	3
Elongation factor 1-alpha 1	3
Mediator of RNA polymerase II transcription subunit 16	3
Protein KIAA0664	3
Uridine-cytidine kinase-like 1	3
Filamin-C	3

Structural maintenance of chromosomes protein 4	3
General transcription factor 3C polypeptide 5	3
Phosphatidylinositol-3,4,5-trisphosphate 5-phosphatase 2	3
Calcium/calmodulin-dependent protein kinase type II subunit delta	3
Plastin-3	3
Integrin-linked protein kinase	3
Eukaryotic translation initiation factor 2 subunit 3-like protein	3
Helicase SKI2W	3
Pyruvate kinase isozymes M1/M2	3
Mini-chromosome maintenance complex-binding protein	3
Insulin-like growth factor 2 mRNA-binding protein 1	3
Cohesin subunit SA-2	3
Zinc finger protein with KRAB and SCAN domains 1	2
HAUS augmin-like complex subunit 8	2
Histone-binding protein RBBP4	2
Intermediate filament family orphan 1	2
FGFR1 oncogene partner	2
Peptide-N(4)-(N-acetyl-beta-glucosaminyl)asparagine amidase	2
ATP-dependent RNA helicase DDX19A	2
Uncharacterized protein KIAA0232	2
MAP kinase-activated protein kinase 5	2
Chaperone activity of bc1 complex-like, mitochondrial	2
Suppression of tumorigenicity 13 (Colon carcinoma) (Hsp70 interacting protein) (Fragment)	2
Cytoskeleton-associated protein 5	2
Tubulin locus 1 (Fragment)	2
Amyloid beta A4 precursor protein-binding family B member 1	2
Glycogen synthase kinase-3 beta	2
Coiled-coil domain-containing protein 85C	2
A-kinase anchor protein 12	2
Apoptosis inhibitor 5	2
Ataxin-3	2
Vacuolar fusion protein CCZ1 homolog	2

Band 4.1-like protein 2	2
Putative methyltransferase METT10D	2
Sorting nexin-2	2
Tryptophanyl-tRNA synthetase, cytoplasmic	2
Tetratricopeptide repeat protein 5	2
Endophilin-A2	2
Phosphatidylinositol-binding clathrin assembly protein	2
Nuclear fragile X mental retardation-interacting protein 2	2
Leucine-rich repeat-containing protein 1	2
Fasciculation and elongation protein zeta-2	2
WD repeat-containing protein 41	2
Heterogeneous nuclear ribonucleoprotein H2	2
Transcription elongation regulator 1	2
V-type proton ATPase catalytic subunit A	2
Protein LSM14 homolog A	2
Actin-like protein 6A	2
Pre-B-cell leukemia transcription factor 1	2
Rap1 GTPase-GDP dissociation stimulator 1	2
RUN and SH3 domain-containing protein 1	2
NudC domain-containing protein 1	2
Heterogeneous nuclear ribonucleoprotein L-like	2
6-phosphofructokinase, muscle type	2
Plakophilin-2	2
Unconventional prefoldin RPB5 interactor	2
Kinesin light chain 4	2
Microtubule-associated tumor suppressor 1	2
Protein misato homolog 1	2
Synaptosomal-associated protein 47	2
Uncharacterized protein	2
Sterile alpha and TIR motif-containing protein 1	2
General transcription factor 3C polypeptide 4	2
Nuclear receptor corepressor 1	2

Uncharacterized protein	2
Far upstream element-binding protein 2	2
Amyloid beta A4 precursor protein-binding family A member 3	2
Zinc finger and BTB domain-containing protein 1	2
Ankyrin repeat and zinc finger domain-containing protein 1	2
Uncharacterized protein OS=Homo sapiens GN=DDX56 PE=4 SV=1	2
Uncharacterized protein OS=Homo sapiens GN=KIAA1217 PE=4 SV=1	2
Probable ATP-dependent RNA helicase DDX5	2
Lysyl-tRNA synthetase	2
Leucine-rich repeat and calponin homology domain-containing protein 1	2
Translation initiation factor eIF-2B subunit delta	2
Histone-arginine methyltransferase CARM1	2
T-complex protein 1 subunit zeta	2
Pleckstrin homology domain-containing family H member 1	2
Histone deacetylase 3	2
Coiled-coil domain-containing protein 22	2
RAS protein activator like 2	2
5'-AMP-activated protein kinase catalytic subunit alpha-2	2
Putative methyltransferase NSUN5	2
Uncharacterized protein	2
AP-4 complex subunit mu-1	2
Histone deacetylase 6	2
Serine/threonine-protein kinase Nek9	2
U1 small nuclear ribonucleoprotein 70 kDa	2
RAD50-interacting protein 1	2
Protein angel homolog 2	2
Selenocysteine insertion sequence-binding protein 2-like	2
Cytoplasmic dynein 1 light intermediate chain 1	2
Stromal cell derived factor 4	2
Valyl-tRNA synthetase	2
Glucosidase 2 subunit beta	2
NudC domain-containing protein 3	2

RAN binding protein 10	2
AP2-associated protein kinase 1	2
Brain protein 16-like	2
Ras GTPase-activating protein-binding protein 1	2
Nephrocystin-4	2
STAT1 protein	2
Bystin	2
Ras GTPase-activating protein-binding protein 2	2
THUMP domain-containing protein 3	2
Signal transducer and activator of transcription 5A	2
Thioredoxin domain-containing protein 5	2
Transcription termination factor 2	2
UPF0668 protein C10orf76	2
5-azacytidine-induced protein 2	2
Regulator of telomere elongation helicase 1	2
Neurochondrin	2
Kelch-like protein 22	2
Polypyrimidine tract-binding protein 1	2
Cytochrome b-c1 complex subunit 2, mitochondrial	2
Uncharacterized protein OS=Homo sapiens GN=DZIP3 PE=4 SV=1	2
DNA-directed RNA polymerase III subunit RPC4	2

translational activator GCN1	4
T complex protein 1 subunit 2	3
SEC16A	3
PAS domain-containing serine/threonine-protein kinase	2
histone acetyl transferase 1	3
histone deacetylase 3	3
cAMP-dependent protein kinase type I-beta regulatory subunit	2
WDR41	2
BIRC6	2
HUWE1	2

Supplementary Table S4: List of primers

Primer name	sequence (5'-)
**GM55**	CGGGTCGACTCATTCCTTCATTCGGAACCGGCCCT
**GM70**	AATTGGAGGAGCAGAAGCTGATCTCAGAGGAGGACCTGA
**GM71**	CCGGTCAGGTCCTCCTCTGAGATCAGCTTCTGCTCCTCC
**GM87**	CCGGACCGGTATCAGAAGAGGCCTGCTG
**GM88**	GCCCTCGAGGCCGTGGTGCTGGTGGTC
**GM93**	CTGTTCCAGGGGCCCGAATTCATGGACGAGAAGGTG
**GM94**	CCGGGGATCCGCTGGAAGTTCTGTTCCAGGGGCCCGAATTC
**GM95**	CCGGGTCGACTTACAGGAAGTAGTCTGGGGTACGAC
**GM96**	GGCCACCGGTATGGCGGCGGCCGAC
**GM97**	CCGGCTCGAGTCAGGCGAGAGACAGAACAGTCAG
**GM98**	GGCCACCGGTATGGACGAGAAGGTG
**GM99**	CCGGGGATCCTTACAGGAAGTAGTCTGGGGTACGAC
**GM103**	CCGGGGATCCATGGCGGCGGCCGAC
**GM109**	CCGGGAATTCCAGCTGAGCCCTGCCGACCTGC
**GM110**	CCGGGTCGACTCAGCCGTGGTGCTGGTGGTC
**GM112**	CCGGAGATCTGGGGGGCCCCAGATTG
**GM131**	CCGGGTCGACTTACCCATGGTGTTGGTGG
**GM142**	GCTTGAATTCATGGTGGTGGATGCGGC
**GM143**	GGATGCGGCCGCTCAGGTCTTTGAGTTGCGG
**GM144**	AATTCAATTGCCCCTGACGCCCCAAG
**GNM271**	GGCCGAATTCGGCTTGAGAGCTTACATCAGAAAACAG
**GNM276**	GGCCGTCGACTCATAGGAATTTCCCATAGATTCTGTGAAGG
**GNM278**	GGCCGGATCCATGCCGCCCGGCAAAG
**GNM279**	GGCCGTCGACTCACAGCGGCTCCAGGTCC
**GNM283**	GGCCGTCGACTCAGGTCTTTGAGTTGCGG
**GNM315**	GGCCCAATTGCTAAAAGACGTTCCATCCTCAGAGC
**GNM316**	GGCCGTCGACTCACGGATTCCAATGTTCTTTTGAAATCC
**GNM336**	GCTTACCGGTATGGTGGTGGATGCGGC
**GNM337**	GGCCGAATTCTCAGGTCTTTGAGTTGCGG
**GNM367**	GGCCGTCGACCTAGCGGCCGTCCTGGG
**GNM380**	GGCCACCGGTATGTTGACATGTAATAAAGCGGGC

Supplementary Figure S1, Maertens G.N.

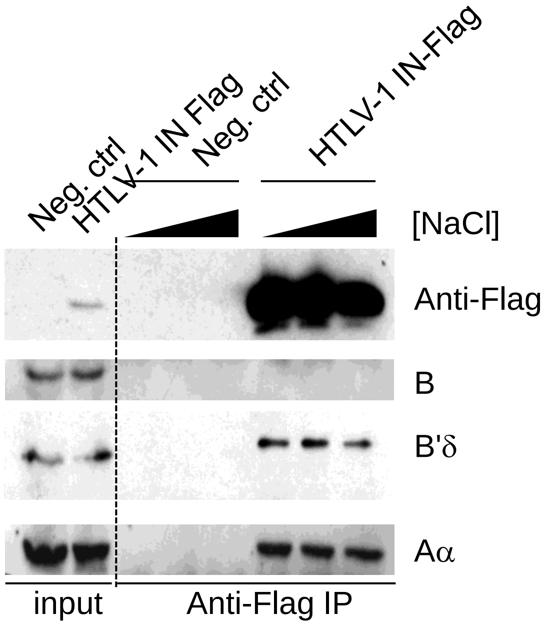

Supplementary Figure S2, Maertens G.N.

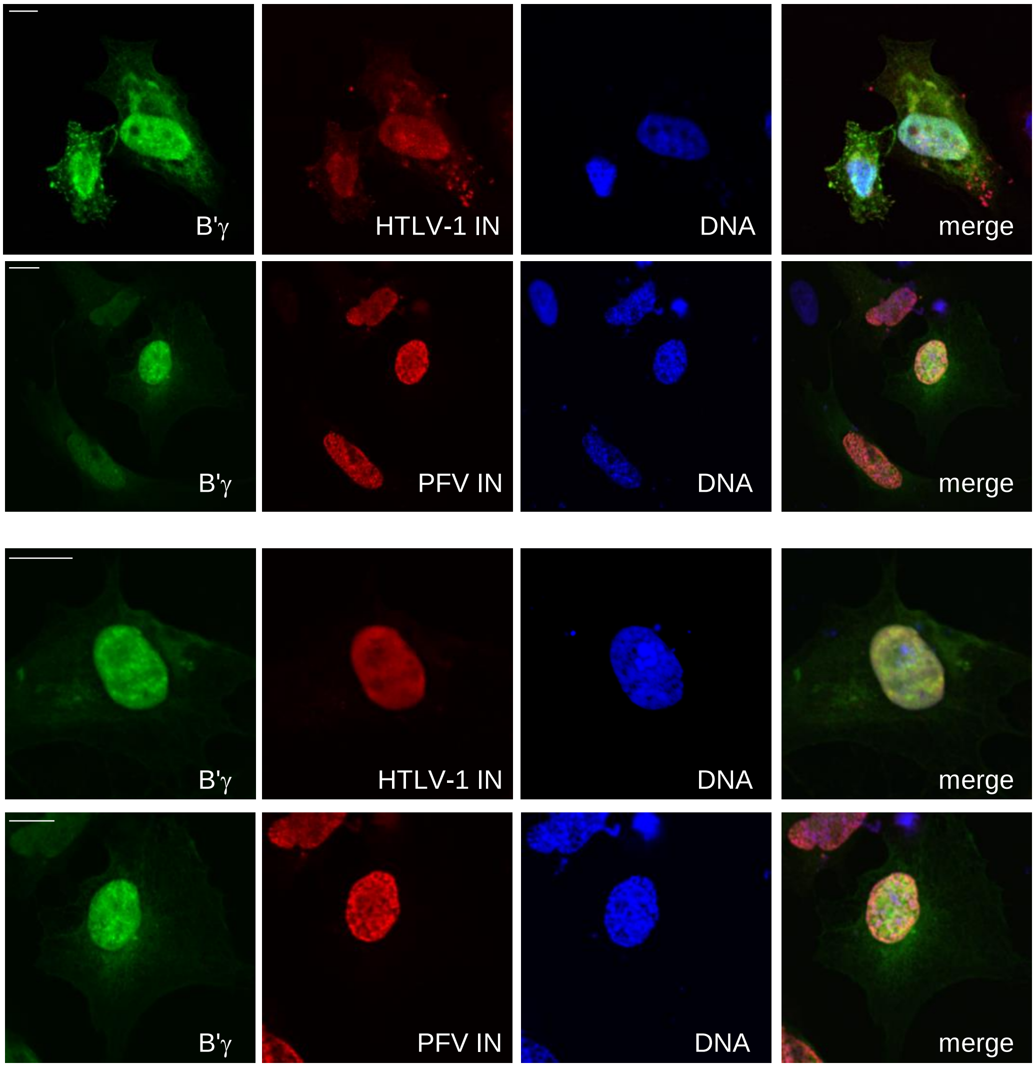

Supplementary Figure S3, Maertens G.N.

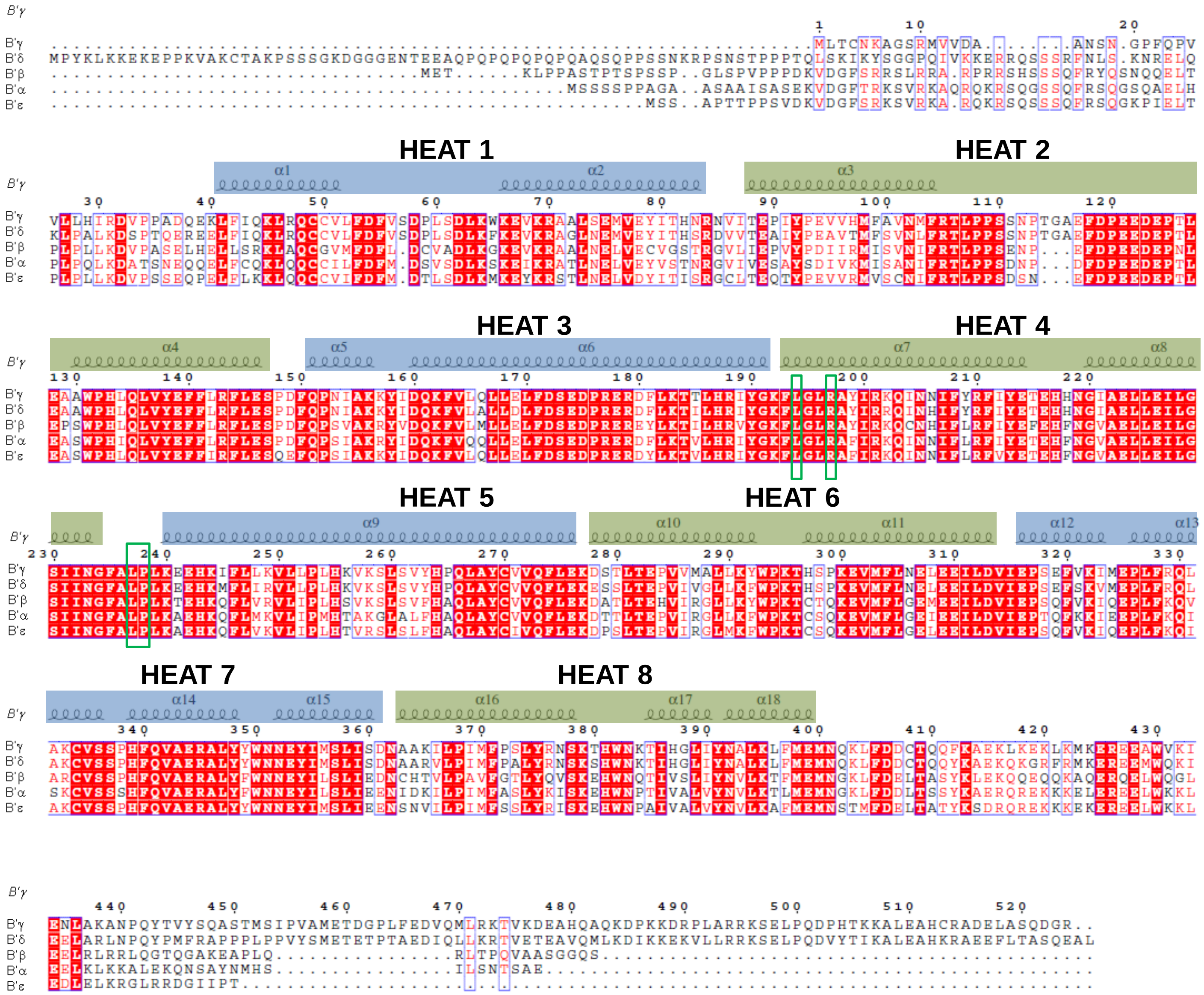

Supplementary Figure S4, Maertens G.N.

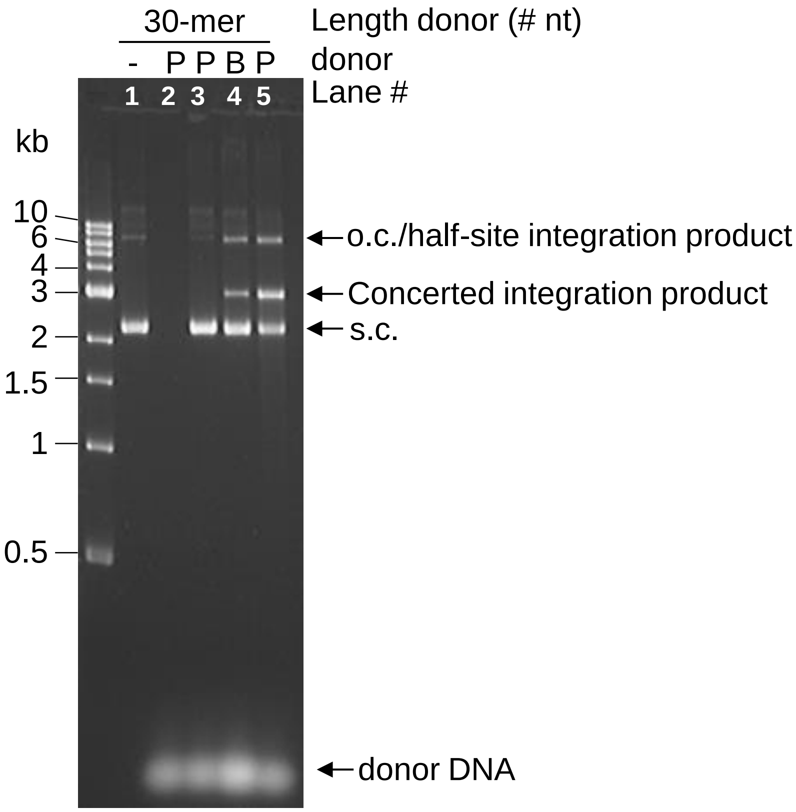

Supplementary Figure S5, Maertens G.N.

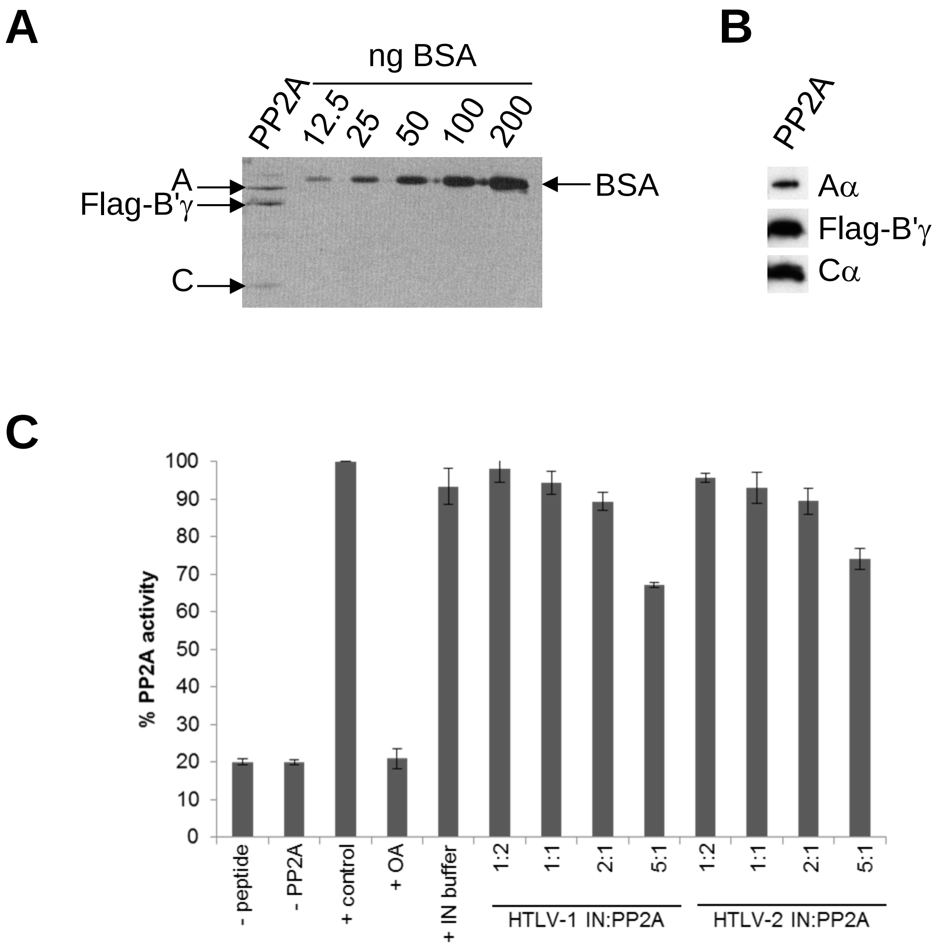

Supplementary Figure S6, Maertens G.N.

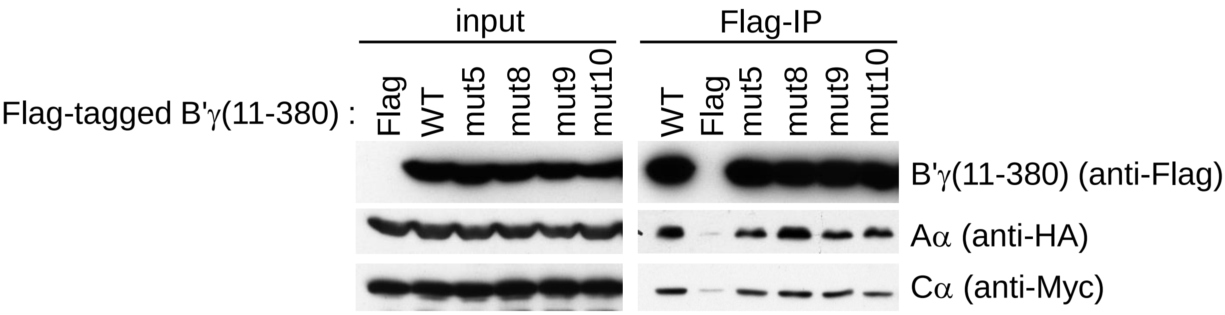

Supplementary Figure S7, Maertens G.N.

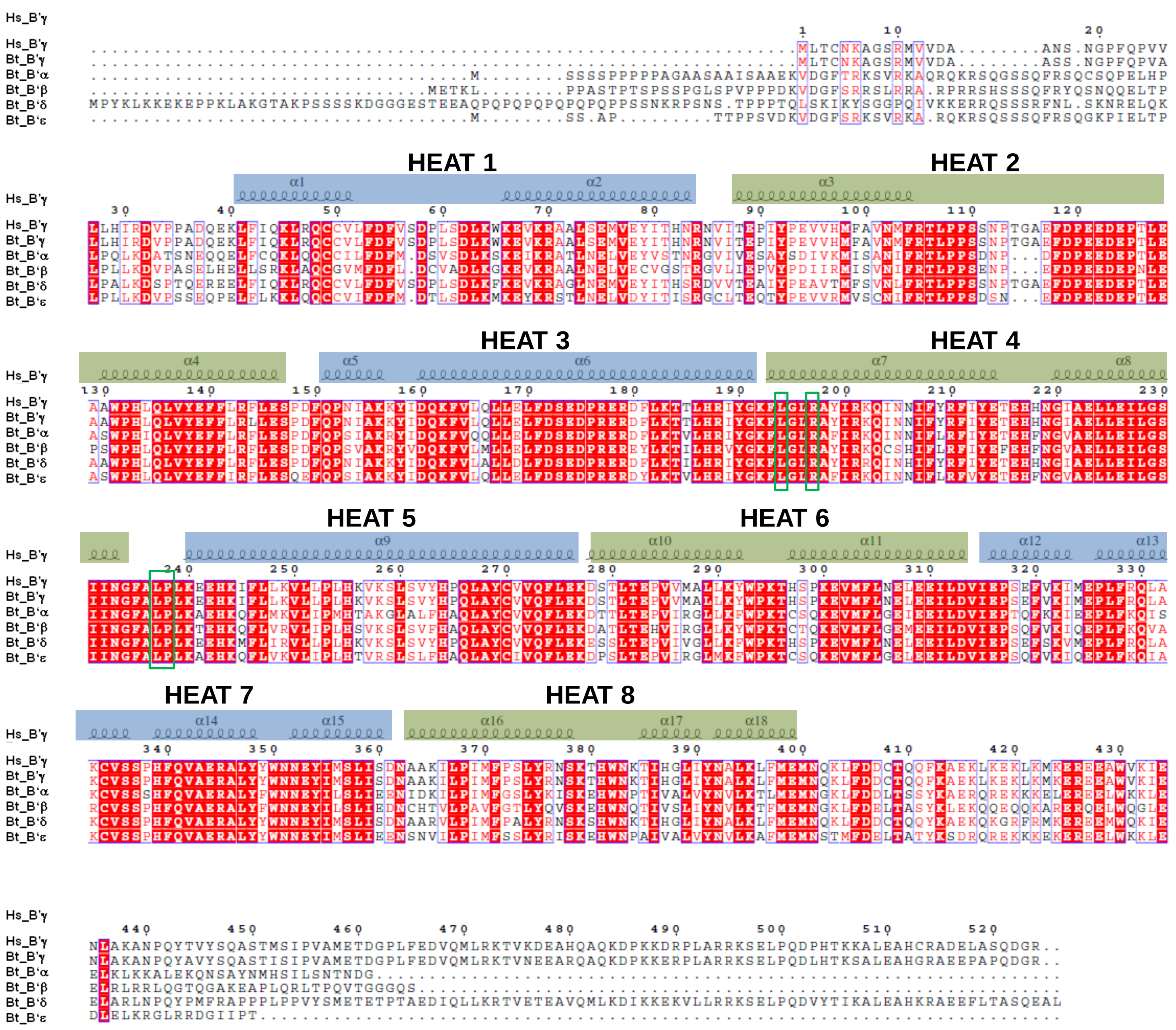

Supplement: SUPPLEMENTARY DATA [file supp_gkv1347_nar-01026-m-2015-File010.pdf]
